# A Fully-Printed Wearable Bandage-Based Electrochemical Sensor with pH Correction for Wound Infection Monitoring

**DOI:** 10.1007/s40820-024-01561-8

**Published:** 2024-11-26

**Authors:** Kanyawee Kaewpradub, Kornautchaya Veenuttranon, Husanai Jantapaso, Pimonsri Mittraparp-arthorn, Itthipon Jeerapan

**Affiliations:** 1 Center of Excellence for Trace Analysis and Biosensor, Prince of Songkla University, Hat Yai, 90110 Songkhla Thailand; 2 Division of Physical Science, Faculty of Science, Prince of Songkla University, Hat Yai, 90110 Songkhla Thailand; 3 Center of Excellence for Innovation in Chemistry, Faculty of Science, Prince of Songkla University, Hat Yai, 90110 Songkhla Thailand; 4 The ijE Electrochemistry for All Laboratory, Prince of Songkla University, Hat Yai, 90110 Songkhla Thailand; 5 Division of Biological Science, Faculty of Science, Prince of Songkla University, Hat Yai, 90110 Songkhla Thailand

**Keywords:** Pyocyanin, Bandages, Wound monitoring, Biosensor, Wearable device

## Abstract

Wearable sensing systems have been designed to monitor health conditions in real-time by detecting analytes in human biofluids. Wound diagnosis remains challenging, necessitating suitable materials for high-performance wearable sensors to offer prompt feedback. Existing devices have limitations in measuring pH and the concentration of pH-dependent electroactive species simultaneously, which is crucial for obtaining a comprehensive understanding of wound status and optimizing biosensors. Therefore, improving materials and analysis system accuracy is essential. This article introduces the first example of a flexible array capable of detecting pyocyanin, a bacterial virulence factor, while correcting dynamic pH fluctuations. We demonstrate that this combined sensor enhances accuracy by mitigating the impact of pH variability on pyocyanin sensor response. Customized screen-printable inks were developed to enhance analytical performance. The analytical performances of two sensitive sensor systems (i.e., fully-printed porous graphene/multiwalled carbon nanotube (CNT) and polyaniline/CNT composites for pyocyanin and pH sensors) are evaluated. Partial least square regression is employed to analyze nonzero-order data arrays from square wave voltammetric and potentiometric measurements of pyocyanin and pH sensors to establish a predictive model for pyocyanin concentration in complex fluids. This sensitive and effective strategy shows potential for personalized applications due to its affordability, ease of use, and ability to adjust for dynamic pH changes.

**Supplementary Information:**

The online version contains supplementary material available at 10.1007/s40820-024-01561-8.

## Highlights

- The first example of a flexible array capable of detecting pyocyanin, a bacterial virulence factor, while correcting dynamic pH fluctuations.
- Developed a screen-printable conductive nanocomposite ink for fabricating the pyocyanin sensor and utilized a polyaniline/carbon nanocomposite as a pH-sensitive film for the pH sensor.
- Customized inks are advantageous in terms of flexible printed sensing array integrated onto a bandage surface, capable of monitoring both pyocyanin and pH levels in wound exudate.

**Supplementary Information:**

The online version contains supplementary material available at 10.1007/s40820-024-01561-8.

## Graphical abstract

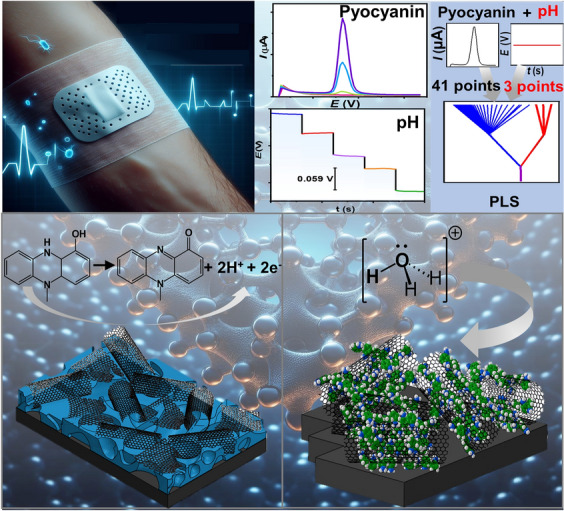

## Introduction

The electrochemical reaction of different chemicals on the electrode material is influenced by the pH of the reaction medium [1]. Pyocyanin [2] and pyoverdine [3] are examples of compounds in which oxidation peak current and peak position are pH-dependent. Although there have been significant advancements in biosensing devices, a limited strategy on wearable platforms has been developed for correcting pH-dependent electrochemical signals during the direct detection of metabolites in real-time. Therefore, the absence of pH correction in electrochemical assessments can lead to considerable distortions in results, compromising the accuracy of the electrochemical measurements.

Wound infection remains a significant healthcare challenge, impacting millions of patients globally [4], due to the intricate demands of diagnosis, monitoring, and management. Recent advancements in electrochemical-based sensors have shown promise in wound monitoring, particularly through the detection of biomarkers such as uric acid [5], lactate [6], and pyocyanin [7]. Pyocyanin is one of the most significant markers for determining wound status. *Pseudomonas aeruginosa* produces pyocyanin during bacterial colonization at the wound, increasing virulence and harming human physiology [8]. The vulnerability of patients can be exacerbated by complications due to wound infections. Prior to the onset of virulent colonization, the release of pyocyanin can indicate the progression of infection before it manifests into severe clinical conditions. The implementation of a wearable sensor would facilitate direct monitoring of wound. Personalized health-status monitoring is increasingly prominent in providing timely alerts for infectious issues beyond the clinical laboratory [9]. Introducing an effective wound monitoring device could lead to shorter hospital stays, fewer visits to healthcare providers, and lower costs associated with laboratory tests for wound assessment and treatment. Therefore, the development of a noninvasive and convenient device for monitoring wound status by analyzing biomarkers is required.

The secretion of pyocyanin from *P. aeruginosa* exhibits variability influenced by factors such as nutrient concentrations, substance presence, and initial bacterial inoculum levels. Elevated pyocyanin levels are a recognized indicator of bacterial growth. Higher bacterial densities lead to increased pyocyanin production, indicating a direct relationship between bacterial concentration and pyocyanin levels [10–12]. Additionally, observing wound infection stages suggests a correlation between the severity of infection and pyocyanin secretion, as burns exhibit significantly higher pyocyanin secretion compared to cases of inflammation [13]. Confirmation of pyocyanin secretion in liquid cultures of clinically isolated *P. aeruginosa* was achieved through electrochemical methods. This evidence underscores the significance of pyocyanin as a biomarker for identifying wound infection [11]. The onset concentration of pyocyanin to indicate the infection varies depending on biological states. For example, within the field of wound healing impairment, normal primary human diploid fibroblasts (HDFs) exposed to pyocyanin (1–50 µM) showed growth arrest and senescence features. Resistance to senescence was observed in HDFs at concentrations of 1–5 µM, while growth cessation was noted in all cells at 10 µM or higher [14, 15].

While there is a demand for a quick method to analyze pyocyanin, there has been a lack of effort in developing sensitive and flexible materials for wearable platforms. In the early days of detecting *P.* *aeruginosa*, analysts often use cell culturing [16], RNA sequencing, spectrophotometry [17], or microscopy [18]. Nevertheless, classical techniques are time-consuming and expensive, necessitating sample pretreatment, specialized equipment, and personnel. For instance, standard wound swab tests in clinical practice require skilled professionals to collect representative samples directly from the wound [19, 20]. Moreover, these often cause delays in obtaining cell culture results in clinical settings, impacting patients and the effectiveness of treatment. Despite plate cultures inoculated from swab samples remaining the gold standard in clinics, bacterial identification through such methods can take up to 24–48 h. Therefore, the development of rapid throughput methods for bacterial infection would be highly desirable.

Pyocyanin’s presence can be detected through electrochemical analysis. Electroanalytical techniques offer several advantages, including rapid detection, simplicity, low cost, and high sensitivity [21]. Electrochemical detection of pyocyanin has been reported, relying on voltammetric methods, including cyclic voltammetry (CV) [22], square wave voltammetry (SWV), and differential pulse voltammetry (DPV) [23]. However, translating traditional sensors into a wearable platform for direct analysis remains a significant challenge. Electrochemical sensors must maintain high sensitivity and stability while in direct contact with epidermally retrieved biofluid, considering the dynamic nature of physiological situations. To achieve success in on-body applications, addressing major analytical challenges is important, particularly concerning uncontrollable parameters such as unpredictable changes in chemistry of biofluids, including fluctuations in pH.

pH is also another important sign for wound infection status. The pH of the wound environment serves as a biochemical marker indicating the healing status. Typically, the pH of intact skin and healing wounds ranges from 4.0 to 6.5, indicating a relatively acidic milieu [24]. This range promotes angiogenesis and epithelization, releases oxygen, and maintains resident commensal bacteria. However, the pH of a chronic wound typically becomes alkaline (pH 7.15–8.90) [25], which can be attributed to bacterial activity and contribute to such issues [26].

**Fig. 1:**
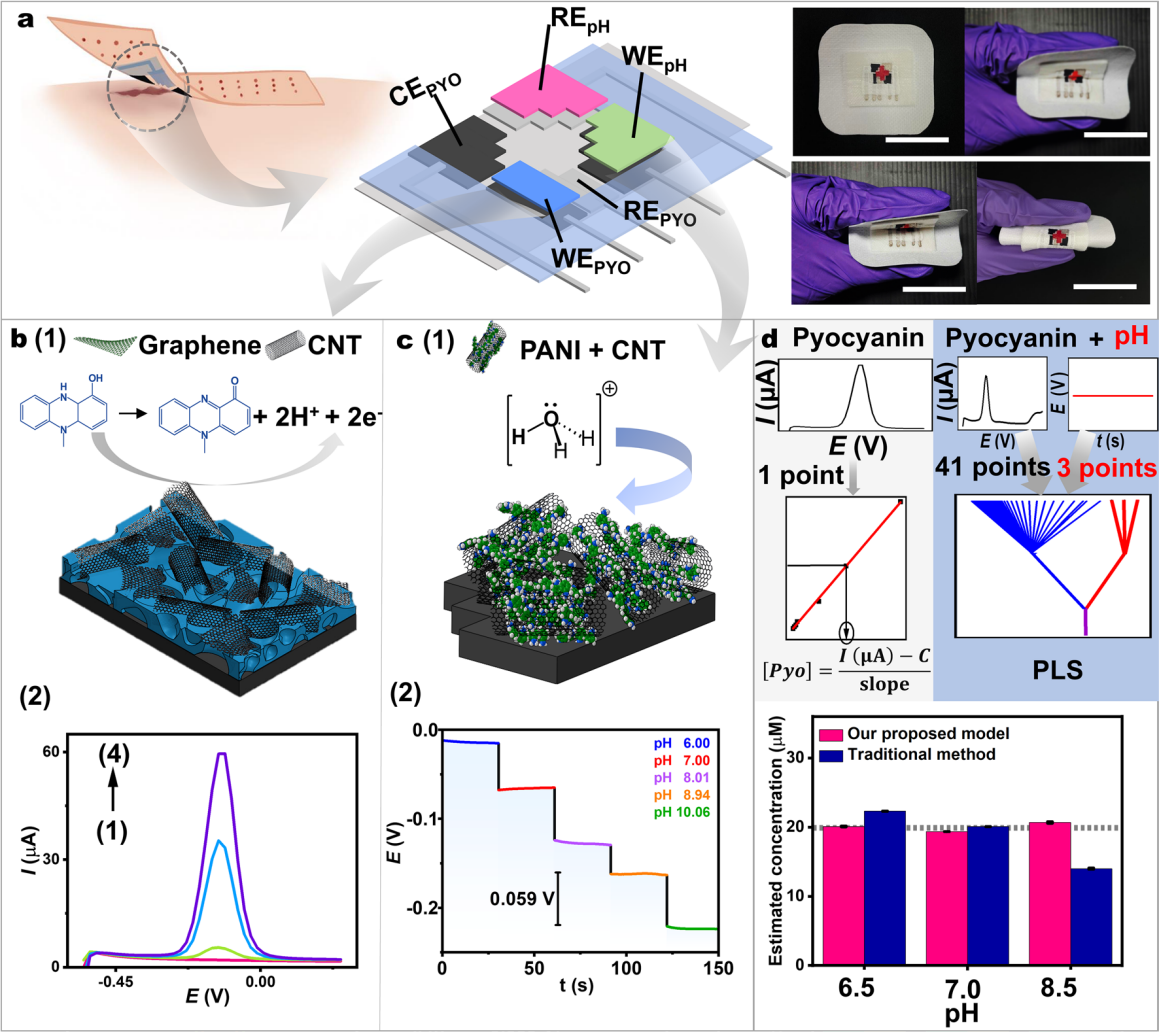
The conceptual presentation of the bandage-based sensing array for determining pyocyanin and pH in wound with a pH-correction system for infection monitoring. **a** Schematic illustration of the bandage-based sensor to directly monitor the wound status and photographs showing the sensors (Scale bar: 2 cm). **b** (1) The electrochemical sensing of pyocyanin on the porous CNT/graphene electrode. (2) Square wave voltammograms (SWVs) for detection of pyocyanin with (1–4) 0, 5, 50, and 100 μM pyocyanin on the porous CNT/graphene electrode. **c** (1) The electrochemical sensing of hydronium ions on the PANI/CNT composite electrode. (2) Potential-time response of the potentiometric pH sensor to various pH solutions. **d** Comparison of predicted pyocyanin concentrations using the traditional regression method (blue) and our proposed method (pink) for pyocyanin concentration analysis at different pH values

Integrating a pH sensor into total analysis systems will enhance accuracy; however, this feature has been overlooked in wearable sensors. While traditional analytical methods can control pH conditions, real-life pH fluctuations in on-body analysis can distort biomarker measurements. Significant progress has been achieved in the field of wearable sensors, including in the development of pH sensors [27–29] and sensors for detecting specific biomarkers, such as pyocyanin [7, 30, 31]. However, existing sensors are very limited, with previous devices capable of detecting either pyocyanin alone or pH alone, such as microneedle-, glove-, and bandage-based sensors [27, 29–32]. Furthermore, comparing the literature on electrochemical sensors employing diverse materials for pyocyanin detection reveals that while some sensors have progressed toward printable electrodes, such as those based on paper or PET (outlined in Table S1), none have integrated pH sensors. To date, there are no reports on strategies coupling pyocyanin and pH sensors in a wearable platform that effectively mitigates the dynamic influence of human biofluid acidity or basicity. This article presents a fully-printed flexible sensing array designed to detect pyocyanin and pH in complex fluids while also compensating for dynamic pH fluctuations. Wearable devices for monitoring wound status are shown in Fig. 1a. Modified nanocomposite materials were integrated onto the surface of a bandage to detect pyocyanin and pH levels simultaneously. To achieve the desired analytical performance of the sensing array, we developed a screen-printable conductive nanocomposite ink for fabricating the pyocyanin sensor and utilized a polyaniline/carbon nanocomposite as a pH-sensitive film for the pH sensor. The combination of both nanomaterial-based sensors resulted in a porous pyocyanin-sensitive transducer and a Nernstian-sensitive pH sensor. To mitigate the impact of pH fluctuations, we developed a mathematical pH-correction system based on partial least squares regression (PLS) to address the issue of pH fluctuations. This wearable design is significant for real-time wound infection screening. It addresses the limitations of existing wound status monitoring methods, which suffer from inaccuracies due to pH effects. By bridging this gap, we aim to create a monitoring array that offers improved accuracy and personalized care for patients.

## Experimental Section

### Fabrication of the Bandage-Based Pyocyanin and pH Array

The pyocyanin electrochemical sensor integrated with the bandage was fabricated by screen-printing a three-electrode system: working electrode (WE_PYO_), pseudoreference electrode (RE_PYO_), and counter electrode (CE_PYO_). A silver conductive ink was printed on a bandage to provide an electrically conductive surface. The carbon-based electrodes were printed by using graphene conductive ink, which served as the working electrode, whereas the counter electrodes were printed by using carbon conductive ink. To enhance the analytical performance of the pyocyanin sensor, the ink for the third layer of WE_PYO_ was prepared by mixing 100 mg of graphene conductive ink, 30 mg of CaCO_3_, and 1 mg of CNTs (diameter: 60–100 nm, length: > 5 μm, and special surface area: 40–70 m^2^ g^−1^) in 1 mL of THF for 90 min in an ultrasonic bath (Elma S30/H—HU193, Germany). Then, the mixture was thoroughly blended using a mixing machine at 2,000 rpm for 30 min (Shashin Kagaku, Kakuhunter, SK-300SII, Japan). After printing, WE_PYO_ electrode was immersed in 0.5 M HCl for 3 h. For RE_PYO_, the Ag/AgCl ink was printed as pseudoreference electrode, followed by drop-casting 6.0 μL of a reference cocktail. This reference cocktail was prepared by mixing 80 mg of PVB with 50 mg of NaCl dispersed in 1 mL of methanol for 30 min in an ultrasonic bath. All electrodes were activated by applying a constant voltage of 1.20 V in 1.0 M Na_2_CO_3_ for 60 s before use.

In parallel, pH sensor was also fabricated by screen-printing a two-electrode system: working electrode (WE_pH_) and pseudoreference electrode (RE_pH_), next to the pyocyanin sensor. A silver conductive ink was first printed on a bandage as the conductive layer for both electrodes. To prepare a CNT-modified ink, 7 g of carbon conductive ink, 30 mg of CNTs, and 490 μL of toluene were mixed using a mixing machine for 30 min at 2,000 rpm. The resulting CNT-modified carbon ink was printed as a working electrode. This printed working electrode was scanned for 20 CV cycles in the aniline solution (i.e., 0.15 M aniline, 1.0 mg mL^–1^ CNTs, 1.0 M sulfuric acid, 0.5 mg mL^–1^ sodium deoxycholate, and 2.0 mg mL^–1^ sodium dodecyl sulfate) with a scan rate of 0.1 V s^–1^ over a range between − 0.30 and 0.85 V to form a PANI/CNT film. Additionally, the Ag/AgCl ink was printed as pseudoreference electrode on the second layer of RE_pH_ followed by dropping 6.0 μL of a reference cocktail (the same as in RE_PYO_) before drying at room temperature. Finally, the electrode areas were defined by using an Ecoflex® layer.

### Electrochemical Studies, Material Characterizations, and Details for Methods and Chemicals

The electrochemical studies involved the utilization of various electrochemical techniques, including SWV, CV, and EIS. All chemical measurements were conducted electrochemically in 0.10 M PBS at pH 7.0, unless stated otherwise. Further details are outlined in the Supporting Information. Detailed information regarding the chemicals, reagents, and preparation methods can also be found in the Supporting Information.

### Traditional Model

To obtain a linear calibration plot of current as a function of pyocyanin concentration, the electrochemical behavior using SWV was studied. First, 9 concentrations of pyocyanin (0, 0.25, 0.5, 0.75, 1, 5, 10, 15, and 20 μM) at pH 7.0 were varied. We measured a sample containing pyocyanin using SWV technique with the pyocyanin sensor without requiring incubation steps. For model validation, validation samples of pyocyanin at different pH values were investigated. For pyocyanin concentration determination, only one data point (which is peak current) was collected, and the pyocyanin concentration was calculated from the calibration curve of pH 7.0 using Eq. (1):

1 $$\left[\text{PYO}\right]=\frac{I - C }{\text{slope}}$$

where [PYO] is pyocyanin concentration (μM), $I$ is peak current (μA), and $C$ is Y-intercept (μA).

### Development of a Partial Least Square (PLS) Regression Model

A model for predicting pyocyanin concentration was constructed using partial least squares (PLS) with nonzero-order voltammetric data obtained from the pyocyanin sensor in different pH solutions and the open-circuit voltage data obtained from the integrated pH sensor. The PLS model was developed using two sets of data: the training data set and the validation data set. Each data set (both training and validation) comprises 41 signal points of current values (obtained from a potential range from − 0.55 to 0.25 V from the pyocyanin sensor) and 3 signal points of pH values (obtained from the pH sensor).

The PLS model was generated using a training data set comprising 9 concentrations of pyocyanin (0, 0.25, 0.5, 0.75, 1, 5, 10, 15, and 20 μM) at 7 pH conditions (6.0, 6.5, 7.0, 7.5, 8.0, 8.5, and 9.0). Each set of experiments was performed with three replicates (*n* = 3). For example, each pyocyanin concentration (e.g., 0.25 μM) underwent SWV detection at various pH values (6.0, 6.5, 7.0, 7.5, 8.0, 8.5, and 9.0). Overall, for one training sample, 41 points of current values at different applied potentials (ranging from − 0.55 to 0.25 V) were obtained from the pyocyanin sensor, while 3 points of potential were obtained from pH sensor. Therefore, a total of 189 samples, each containing 41 data points, were used for multivariate analysis using the PLS technique in Origin 2022.

For the validation data set, 3 pyocyanin concentrations (5, 10, and 20 μM) at 3 pH values (6.5, 7.0, and 8.5) were varied, resulting in 27 samples (*n* = 3). The SWV was performed for pyocyanin sensor while potentiometric measurement was carried out for pH sensor. The data points obtained from SWV technique (41 data points per sample) and potentiometric measurement (3 data points per sample) were recorded and used to validate the established PLS regression model.

### Pyocyanin Determination from Bacterial Culture

*P. aeruginosa* ATCC27853 was used as the starting culture for pyocyanin pigment production. Briefly, 3–5 colonies of *P. aeruginosa* were cultured in 1X Mueller–Hinton broth (MHB). The inoculated broth was incubated at 37 °C while shaking at 150 rpm for 4–5 h to reach the log phase of growth. After incubation, the cell density was adjusted to a 0.5 McFarland standard, which corresponds to approximately (~ 1 × 10^8^ CFU mL^–1^). The culture was then diluted in MHB to obtain the final desired concentrations of bacteria. Additionally, artificial wound solution and MHB were prepared (2X concentration). These solutions were then mixed at a 1:1 ratio. This mixing resulted in a final solution with a 1X concentration for both MHB and artificial wound fluid. After that, bacterial culture broth at each desired concentration was added to the mixed solution (1:1), resulting in the wound solution being at a 0.5X concentration in the culture plate. The culture plates were then incubated at 37 °C under aerobic conditions for various growth times, specifically 0, 8, 16, 24, 36, and 48 h. After incubation, the culture was pipetted into a culture chamber. Bacterial growth and pyocyanin production were assessed by measuring the absorbance at 600 and 520 nm, respectively [33, 34].

Additionally, 100 μL of culture was taken and subjected to a serial dilution (1:10) in a 0.85% NaCl solution. Following this, 5 μL of the diluted bacterial suspension was dropped onto Tryptic Soy Agar (TSA). The TSA plates were incubated at 37 °C for 20 h under aerobic conditions. The resulting colonies were counted, and the number of colony-forming units (CFU) was determined and expressed as CFU mL^–1^. In parallel, bacterial culture broth was analyzed using an electrochemical technique. This involved utilizing a portable wireless device to detect the pyocyanin concentration.

### Biocompatibility Test Using MTT Assay

The biocompatibility of sensor materials was assessed via an MTT assay using L-929 cells [35], with percent cell survival (viability) calculated from spectrophotometric absorbance data (see Supporting Information for details).

## Results and Discussion

### Wearable Bandage-Based Sensing Array with pH-Correction System Concept

The integration of real-time monitoring offers significant potential to speed up wound treatment [32]. Infections can hinder wound healing and lead to severe complications [36]. This emphasizes the need for prompt detection to facilitate timely treatment and prevent exacerbation. Our proposed solution developed effective materials for creating a wearable sensing tool, aiming to reduce reliance on highly skilled healthcare professionals, while eliminating delays in critical treatments. We employed a bandage as a substrate for screen-printing flexible electrodes aimed at detecting infection markers. We developed two working electrodes (WEs) for monitoring pyocyanin and pH, utilizing a porous CNT/graphene electrode and a PANI/CNT-based electrode, respectively, due to their unique electrochemical and mechanical advantages. The counter electrode (CE) and reference electrode (RE) were screen-printed using graphene conductive ink and Ag/AgCl ink. These screen-printed sensors demonstrate flexibility and cost-effectiveness in production, along with rapid and sensitive sensing capabilities, making them well-suited for practical on-body applications. Electrochemical sensing mechanisms for detecting pyocyanin in wound exudate are presented in Fig. 1b(1). The sensor is specifically engineered to enable the direct electrochemical analysis of pyocyanin by facilitating its transfer from wound fluids onto the electrode surface of the bandage. The redox-active nature of pyocyanin allows the molecule to undergo oxidation on a suitable material surface, as observed from the SWV response (Fig. 1b(2)). However, it is challenging to develop an electrocatalytic surface that enhances conductivity and electron transfer capability while maintaining mechanical resilience on a wearable substrate. The modification requires carefully engineering the material’s morphology and chemical composition to achieve a sensitive and flexible sensor. In our work, we have developed a porous structure for the working electrode of the pyocyanin sensor. This design enhancement aims to improve the surface area, enabling continuous species transport through a porous network [37]. Developing porous CNT/graphene electrode composites further enhances sensitivity and selectivity.

Additionally, an integrated bandage-based pH sensor was utilized to evaluate wound status. The printed electrode was modified with a polyaniline (PANI)/CNT composite, serving as both the pH-sensitive polymer and conductive material. The pH sensor is important for supporting a model to mitigate issues arising from changes in pH. This is significant because such pH changes can affect the electrochemical signal when detecting targets, such as pyocyanin. In Fig. 1c(1), the PANI/CNT composite was deposited onto the working electrode area to create a PANI-based conductive membrane. The PANI-based transducer on the surface of the electrode acts as a layer for a reversible protonation-deprotonation process. Upon protonation/deprotonation of hydronium ions from the wound fluid, the resulting interaction at the interface between the biofluid and the sensitive electrode induces a voltage alteration. The change in electrode potential can be recorded relative to the reference electrode, yielding the potentiometric signal. Figure 1c(2) illustrates sensor responses at varying pH levels ranging from 6.0 to 10.0.

To introduce the need for developing the integrated array and model to correct the pH effect, pyocyanin concentrations predicted from the traditional linear regression method were compared to those predicted from our proposed method, using pyocyanin samples at different pH values—representing acidity, neutrality, and basicity (Fig. 1d). In the traditional model, the resulting concentration is predicted by referencing the standard calibration curve of pH 7.0 following Eq. (1). This traditional approach employs zero-dimensional data, treated by linear regression between peak current at one particular voltage and the concentration of pyocyanin. In contrast, our proposed method involves calculating the concentration of samples using more data points—specifically, 41 SWV data and 3 potentiometry data—with pH correction using the PLS model. The findings confirmed that the traditional method (without the integrated pH sensor) overestimated the pyocyanin concentration by 11.6% at pH 6.5 and underestimated by 30.0% at pH 8.5, whereas our developed system could estimate pyocyanin concentration more accurately. Applying linear regression, a univariate analysis, may seem simple; however, this approach can result in errors as it ignores many other relevant current values, especially in diverse pH solutions. When integrating the pH sensor with the pyocyanin sensor, the pH sensor can serve to double-check and enhance accuracy. Understanding the pH effect and its implications on analytical performance is crucial for developing robust analytical methods that can operate effectively under varying pH conditions, ensuring accurate results without requiring the pretreatment step of pH adjustments, which is the existing approach in traditional benchtop analytical chemistry but is too challenging in on-demand or on-body systems. If there is no pH-correction system, in cases where either no pyocyanin is present or the pyocyanin concentration is too low for observation via the voltammetric sensor alone, relying solely on a voltammetric sensor to observe the oxidation peak of pyocyanin at unknown and dynamically fluctuating pH levels will be an issue for analytical purposes. In such instances, a pH sensor becomes essential to provide additional vital information about biological situations. Therefore, in addition to developing a pyocyanin-sensitive electrode, it is essential to create material arrays that integrate a pH-sensitive electrode to achieve an accurate response converging to the true value of pyocyanin concentration. This ensures a reliable diagnosis of wound status in an on-skin total analysis system. The details of developing the correction model will be discussed in Sect. 3.5.

### Characterizations and Study of Electrochemical Behaviors of Bandage-Based Porous CNT/Graphene Electrodes

Developing porous nanomaterials is crucial for enhancing electroanalytical performance in pyocyanin oxidation. We improved them by developing a flexible printed porous material. A screen-printable ink was formulated by blending conductive nanocomposite, binder, and organic solvent with CaCO_3_ porogen. This porogen, insoluble in selected organic media, could dissolve in an acidic aqueous solution. Through chemical etching, the porogen was selectively removed, generating CO_2_ gas to open the pore mouths. This facile concept of porogen removal, fabricable in non-harsh conditions or moderate temperatures, allows direct fabrication on a soft wearable substrate, facilitating the introduction of pores into the printed nanomaterial. This achieves the desired flexible and porous electrode on the bandage.

**Fig. 2:**
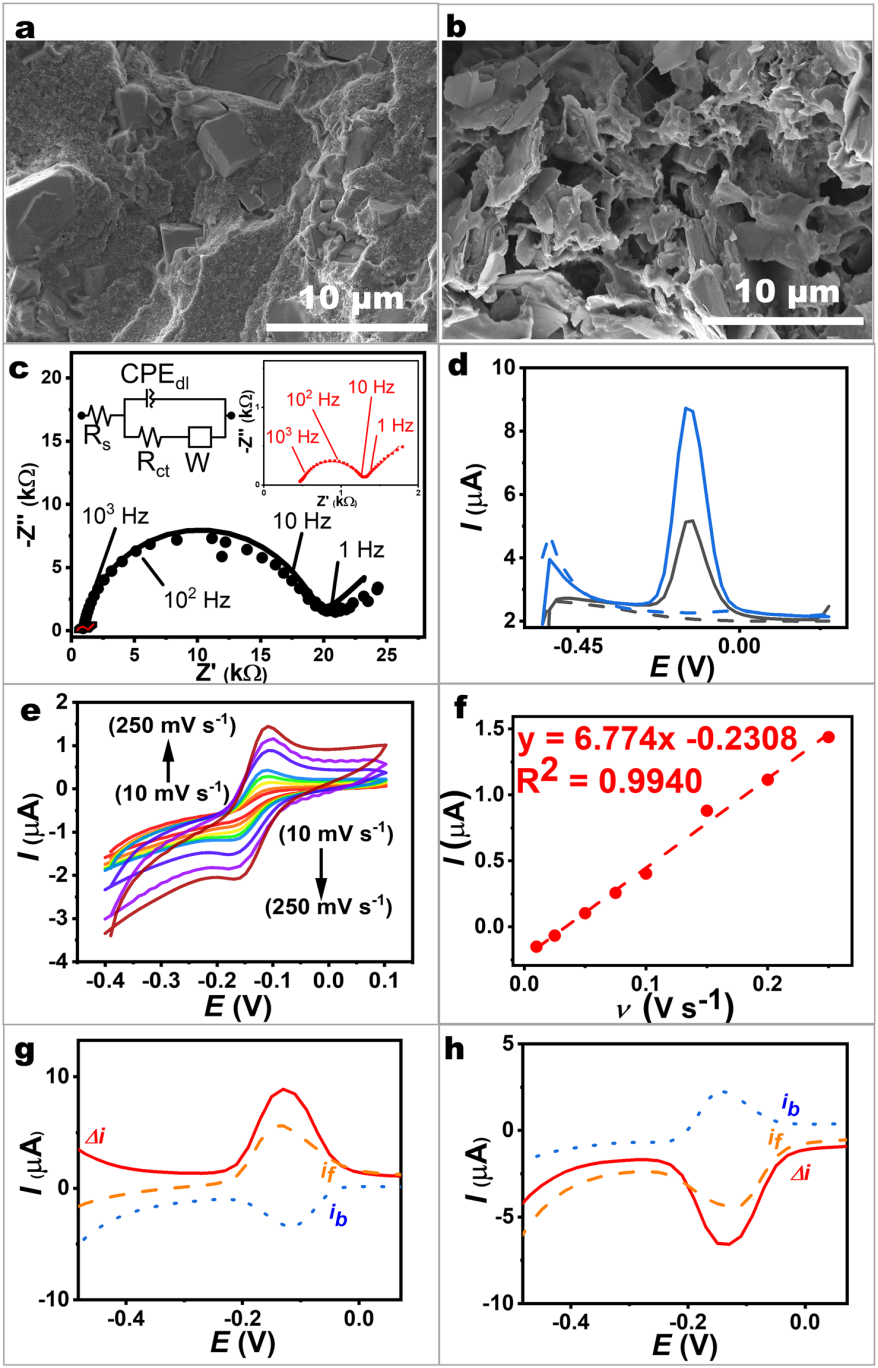
Electrochemical performances and material characteristics of printed bandage-based electrodes. **a, b** SEM images of printed CNT/graphene electrodes: **a** before removing CaCO_3_ and **b** after removing CaCO_3_. **c** EIS plots using (black plots) unmodified material electrodes and (red plots) printed porous CNT/graphene-based electrodes. EIS was conducted in a frequency range of 1 × 10^−1^–1 × 10^4^ Hz and an amplitude of 10 mV. The inset depicts the equivalent circuit used for data fitting. The solid lines represent the fitted graphs. **d** SWVs (black plots) from unmodified material electrodes and (blue plots) printed porous CNT/graphene-based electrodes in 10 µM pyocyanin. Blank responses are depicted as dashed lines. **e** CVs obtained from the porous CNT/graphene electrode in the presence of 10 µM pyocyanin at different scan rates. **f** Plots of peak current densities as a function of the scan rate. **g** SWV from the oxidation of 10 µM pyocyanin on a porous CNT/graphene electrode. The signal (*Δ*_*i*_) and its forward (*i*_*f*_) and backward (*i*_*b*_) components are depicted. **h** SWV for the reduction direction

To examine the composite material comprising base ink, CaCO_3_, and CNTs, scanning electron microscopy (SEM) was used to analyze the electrode surface structure. Figure 2a shows a uniform distribution of CaCO_3_ within the ink on the electrode surface. After removing the CaCO_3_ porogen, as depicted in Fig. 2b, numerous pores emerge uniformly. These images clearly confirm that the removal of the porogen increased the porosity of the electrode, resulting in a larger active surface area. Additionally, optical microscopy was also employed to further assess the physical features of the porous CNT/graphene electrode. Such examination revealed differences in the electrode’s physical features before and after the removal of CaCO_3_ (as shown in Fig. S1). Prior to the removal process, the electrode exhibited CaCO_3_ adhesion, indicated by the presence of dots on its surface (Fig. S1a). Following the removal of the porogen through immersion in HCl solution, an inspection revealed the absence of CaCO_3_ white spots on the electrode surface (Fig. S1b). This printable porosity-generation method effectively addresses the challenge of limited area on miniaturized printed electrodes.

To assess the impact of material engineering, contact angle measurements were also conducted (Fig. S2). The unmodified electrode has a smooth surface, forming rounded water droplets with an approximate contact angle of 58° (Fig. S2a), indicating a hydrophilic characteristic. In contrast, the porous CNT/graphene electrode before undergoing Na_2_CO_3_ electrochemical activation shows higher surface roughness with a contact angle of 84° (Fig. S2b), attributed to the presence of CNTs and the porous structure [38, 39]. Subsequent electrochemical treatment of the porous material with a Na_2_CO_3_ solution under anodization results in flatter water droplets with a smaller contact angle of 81° (Fig. S2c). This treatment effectively removes impurities while improving the material surface’s hydrophilicity [40].

To investigate the material composition of the electrode surface and analyze the elemental composition of the electrode material, we conducted elemental distribution analysis. Our focus was on examining printed CNT/graphene electrodes before and after CaCO_3_ removal, as illustrated in spectra in Fig. S3a, b. The results revealed a clear change in the calcium (Ca) content before and after removal of the CaCO_3_ template. Prior to removal, the template had a Ca content of 13%, which subsequently disappeared after removal. This evidence confirms that the dissolution process completely eliminates the CaCO_3_ template, resulting in a porous structure on the surface of the screen-printed material, which aims to facilitate the transport of interesting species.

Furthermore, to observe the process of porogen removal and pore generation, the comparison of the electrochemical performances between printed CNT/graphene composites before and after CaCO_3_ removal was conducted. The electrode before CaCO_3_ removal showed an oxidation peak of pyocyanin at approximately − 0.13 V with an anodic current of 7.20 µA (Fig. S4). Following CaCO_3_ removal, the porous CNT/graphene electrode exhibited a higher oxidation peak of pyocyanin, with an anodic current of 8.62 µA. This increase in catalytic current for pyocyanin oxidation is attributed to the improved electrode area and pore opening.

Cyclic voltammetry was utilized to determine the surface area and electron transfer performances of the unmodified electrode and our printed CNT/graphene electrodes (prior to and following the removal of the CaCO_3_ porogen), employing a standard redox solution (Fig. S5). The resulting *I*_pa_ and *I*_pc_ values from these electrodes showed a proportional correlation with the square root of the scan rate, indicating a diffusion-controlled characteristic. The engineered nanocomposite ink has the potential to enhance favorable electron transfer and active surface area, as inferred by the observation of smaller peak-separation potential and increasing peak currents. Estimation of the active electrochemical surface area using the Randles–Sevcik relationship (refer to Note S1) revealed that our developed printed materials demonstrated a significantly higher electroactive surface area compared to unmodified electrodes (which displayed a small active area of 5.7 × 10^–4^ cm^2^). Subsequent treatment of the printed CNT/graphene electrode with acid etching increased the electroactive surface area to 0.13 cm^2^, indicating a high roughness factor of 130%. Such an enhanced active electrode area is approximately 22,500% and 21% higher compared to the active surface area of the unmodified electrode and the nonporous CNT/graphene electrode, respectively.

We also explored the porosity of the printed porous CNT/graphene-based surface by examining the accessibility of electrolyte ions, comparing it with the unmodified material and the nonporous CNT/graphene material. This analysis involved recording CV curves in a KCl solution (Fig. S6). With our porous CNT/graphene-based material, the CV profiles consistently showed a box-like shape across scan rates ranging from 10 to 250 mV s^−1^, indicating a highly porous structure with a large surface area that facilitates ion movement at the electrode–electrolyte interface. The porous structure enhances the penetration and diffusion of electrolyte and analyte molecules, thereby promoting efficient electrochemical analysis. Notably, the capacitance increased from 8.34 to 38.4 µF cm^−2^ (at a scan rate of 10 mV s^−1^) for the unmodified ink electrode and the porous CNT/graphene electrode, respectively (refer to Fig. S6a(3), b(3), and Note S2). As the scan rate increased, the capacitance value decreased (Fig. S6b). The decrease in capacitance at elevated scan rates is attributable to the constrained duration for charging/discharging processes to occur at the electrode interface. Nevertheless, at a rapid scan rate of 250 mV s^−1^, porous CNT/graphene maintained 72% of its specific capacitance relative to the slower rate (10 mV s^−1^), affirming the material’s porosity on the active surface [41].

Further investigation utilizing electrochemical impedance spectroscopy (EIS) was conducted to analyze the printed porous carbon-based material, utilizing alternating current with a small potential perturbation (10 mV) (see Fig. 2c). The EIS experiments were carried out in a solution containing 2.5 mM potassium ferricyanide and 2.5 mM potassium ferrocyanide dissolved in 0.1 M KCl. Nyquist plots were generated across frequencies ranging from 100 mHz to 10 kHz. Through EIS and the fitted model, various electrochemical phenomena including the capacitive electric double layer, faradaic reactions of standard redox species, and diffusion dynamics across varied time domains were characterized. We observed a semicircle behavior in the high-frequency region (above approximately 1 − 5 Hz, corresponding to time domains shorter than 0.2 − 0.8 s) for both electrodes. The EIS characteristic parameters of the porous CNT/graphene and unmodified materials are shown in Table S2. The semicircle of the porous CNT/graphene electrode demonstrated a smaller charge transfer resistance (*R*_ct_) of 0.788 kΩ compared to the unmodified electrode, which showed *R*_ct_ of 18.2 kΩ, suggesting enhanced electrochemical performance attributed to its specialized structure and printed conductive nanocomposites. Moreover, Bode modulus values further confirmed that the porous CNT/graphene electrode exhibited lower modulus (ranging from 1.2 to 1.8 kΩ) compared to the unmodified electrode (ranging from 13.4 to 24.5 kΩ) within the frequency range of 100 mHz to 40 Hz (Fig. S7). This difference underscores the role of modifications, such as incorporating carbon nanotubes and creating a porous structure, in reducing charge transfer resistance. These modifications increase the available active area for charge transfer and enhance the electrode’s conductivity. This enhancement can be attributed to the formation of a porous network as confirmed by imaging analysis. In contrast, the absence of such features in the unmodified material limited its active surface area, and the absence of nanocatalysts in the printed surface, resulting in higher resistance.

The electrochemical behaviors of a bandage-based porous CNT/graphene electrode were investigated for the detection of pyocyanin. Square wave voltammetry (SWV) was employed to study the oxidation of the analyte in a standard solution. In Fig. 2d, a comparison between the pristine ink electrode and the porous CNT/graphene electrode after porogen removal is shown. A small oxidation peak of pyocyanin at around −0.13 V with an anodic current of 5.17 µA was observed on the pristine ink electrode (black plot). Remarkably, the addition of the porous CNT/graphene electrode (blue plot) significantly enhanced the oxidation current of pyocyanin, reaching 8.62 µA (an increase of ~ 2 times). This enhancement underscores the beneficial impact of incorporating CNT/graphene materials and porosity in amplifying the electron transfer kinetics, thereby enhancing the sensitivity and efficiency of pyocyanin detection.

To explore the electrochemical behavior of the electrocatalytic porous CNT/graphene electrode toward pyocyanin oxidation, we conducted CV. As observed in Fig. 2e, peak currents increased with higher scan rates. The anodic (*I*_pa_) and cathodic peak (*I*_pc_) currents, corresponding to the oxidation and reaction of pyocyanin, respectively, exhibited an *I*_pa_*/I*_pc_ ratio of 1.09 for the porous CNT/graphene electrode. This finding confirms reversible reactions on the printed porous surface. With only a slight shift of around 10 mV in anodic peak potential as the scan rate increased from 10 to 250 mV s⁻^1^, the formal potential remained nearly independent of the scan rate within this range, confirming facile charge transfer kinetics on the electrocatalytic porous CNT/graphene electrode. Anodic peak currents showed direct proportionality to the scan rate, as depicted in Fig. 2f. The surface coverage of pyocyanin on the porous electrode could be estimated to be 1.8 × 10⁻^11^ mol cm⁻^2^ (refer to Note S3).

Beyond CV, SWV was chosen to enhance electroanalytical performance. The distinct peak observed in the voltammogram of pyocyanin emerges at − 0.13 V, as illustrated in Fig. 2g. The presence of both forward (anodic, *i*_*f*_) and backward (cathodic, *i*_*b*_) waves indicate the quasi-reversible nature of the pyocyanin redox reaction. To validate these findings, we reversed the scan direction. The result in Fig. 2h confirms that the forward signal manifests as a reductive current when the scan commences at 0.25 V and sweeps toward the left side (i.e., toward the reduction zone). This observation aligns with the previously presented CV results (Fig. 2e).

**Fig. 3:**
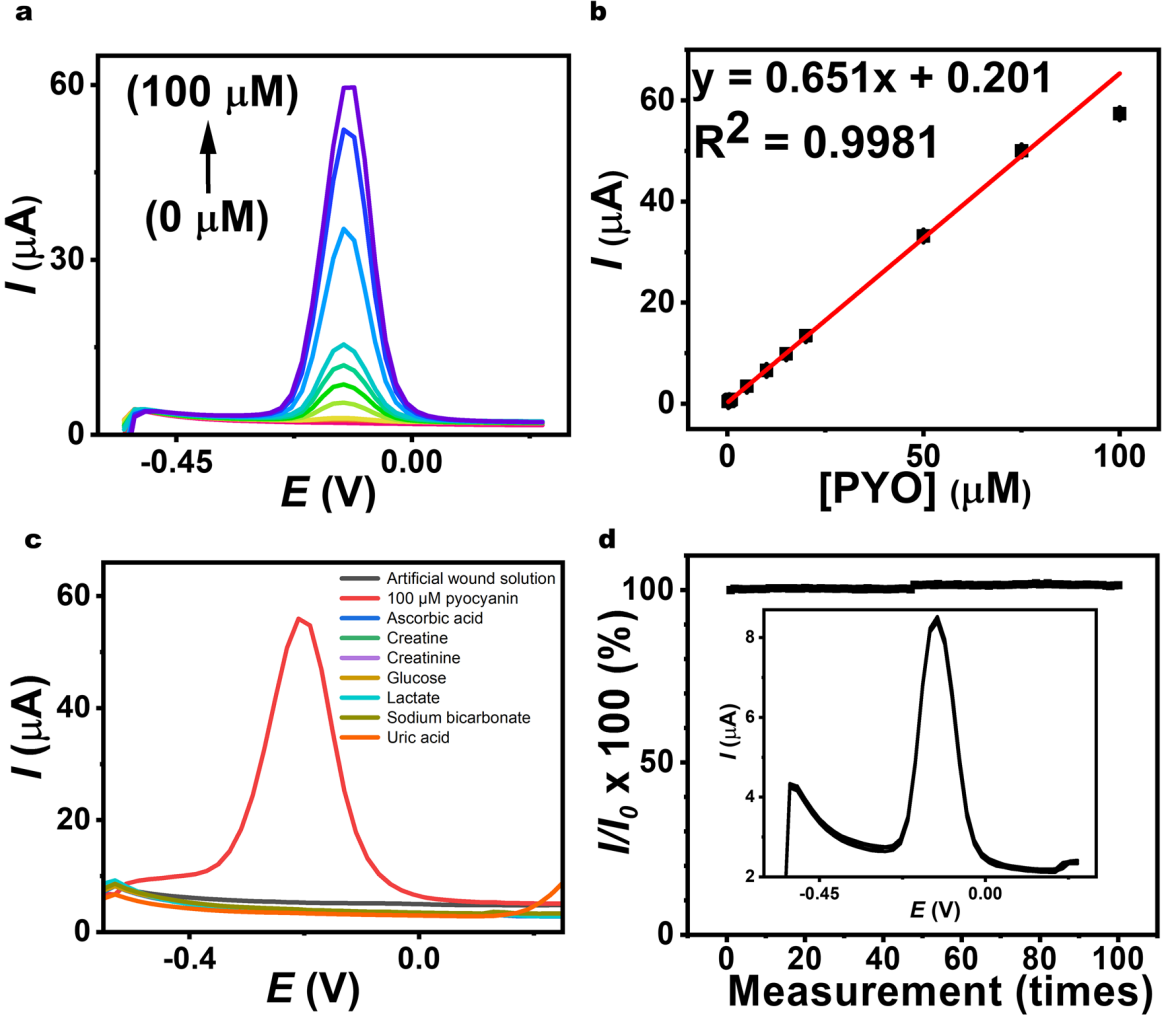
SWV measurements conducted on a porous CNT/graphene electrode. **a** SWVs on a porous CNT/graphene electrode for different concentrations of pyocyanin (*n* = 3). **b** The corresponding calibration plot. **c** Interference tests using SWV. All interferences were prepared in an artificial wound solution. **d** The repetitive SWV curves obtained at the porous CNT/graphene electrode in 10 µM pyocyanin

Pyocyanin solutions with varying concentrations were analyzed using SWV technique. Figure 3a displays the voltammetric responses across pyocyanin concentrations up to 100 µM, indicating low background current within this wide concentration range. An increase in the anodic peak current at − 0.13 V was observed with increasing pyocyanin concentration, while no shift in the oxidation potential was detected. This absence of peak potential shift suggests minimal kinetic limitation, due to the effective surface of the printed porous material, enabling efficient transport of pyocyanin molecules and its oxidation reaction. The extracted peak current data, as shown in Fig. 3b, were used to establish a calibration curve exhibiting linearity over a range of 0.25–100 μM pyocyanin. The linear regression equation, *I*_pa_ (µA) = (0.6513 ± 0.009)*C*_pyocyanin_ (µM) + (0.201 ± 0.049), displayed good linearity (*R*^2^ = 0.9981), with a detection limit of 0.22 μM (3 × SD/sensitivity; where SD represents the blank deviation). The enhanced performance can be attributed to the synergistic effects achieved by the porous CNT/graphene electrode, facilitating efficient electrocatalytic signal transduction and contributing to the transducer’s enhanced performance. Notably, this wearable bandage-based electrochemical sensor distinguishes itself from other works listed in Table S1 by integrating two types of sensor systems. These flexible sensors detect pyocyanin concentration and pH values, reducing errors associated with pH variations.

In real wound exudate, electrolytes and metabolites such as uric acid, urea, creatinine, ascorbic acid, and glucose are commonly found, potentially interfering with pyocyanin sensors. Hence, assessing the impact of these agents is crucial to ensure the selectivity of the developed pyocyanin sensing material. In this study, various possible interfering agents—0.072 mM ascorbic acid, 0.4 mM creatine, 0.6 mM creatinine, 11.8 mM glucose, 33.4 mM lactate, 44 mM sodium bicarbonate, and 1.5 mM uric acid—were tested in an artificial wound solution, with concentrations double those reported in the literature [42–44]. This setup aimed to demonstrate that even under elevated interference levels, there was no problematic impact on the peak current for pyocyanin detection. Figure 3c shows that such tested species (i.e., artificial wound exudate and the fluid with several common interfering agents) did not affect pyocyanin determination. Despite uric acid and ascorbic acid being possible electrochemical interferences in biological fluids, no peaks associated with these substances were detected within the scanning range of − 0.55 to 0.25 V. This is attributed to the fact that easily oxidized compounds, such as uric acid and ascorbic acid, have oxidation peaks separate from that of pyocyanin, enabling differentiation. Our porous CNT/graphene electrode exhibits excellent selectivity tailored for pyocyanin detection using SWV, highlighting its robust performance in complex exudate.

Examining the stability of the wearable sensor is also crucial for sustained performance during continuous monitoring. Repeatability studies were conducted on the porous CNT/graphene electrode using the SWV technique with 10 µM pyocyanin. As shown in Fig. 3d, the current changes slightly after 100 measurements, with a relative standard deviation (RSD) of 0.59%. This indicates favorable long-term operational stability of the printed porous CNT/graphene material, confirming the sensor’s durability for prolonged measurements. Additionally, the stability of the pyocyanin sensor was assessed by measuring pyocyanin at pH values of 6.0, 7.0, 8.0, and 9.0 and the experiment repeated across three cycles of ascending and descending pH sequences. As shown in Fig. S8, current changes were minimal throughout the experiment. This stability highlights the sensor’s potential for monitoring in fluctuating pH conditions typical of wound environments.

Variations in the pH of pyocyanin-containing fluids may impact the redox output on the printed porous material. We investigated the effect of different pH levels on pyocyanin electrooxidation using the printed porous CNT/graphene electrode within the pH range of 5.0 to 9.0. Figure S9a illustrates the systematic analysis of the effect of pH variation in the pyocyanin-containing medium on electrochemical response. The data reveal an observable trend: as the pH of the pyocyanin-containing fluids increased, the SWV peaks shifted linearly toward more negative values, with a slope of − 0.054 mV pH^−1^ on the porous CNT/graphene electrode (Fig. S9b). This observation, evident from the near-Nernstian slope, confirms that pyocyanin oxidation follows a two-proton, two-electron reaction. Given the significant pH dependence of the pyocyanin oxidation reaction, implementing a pH-correction system is crucial for maintaining measurement accuracy.

### pH Monitoring with Integrated Wearable PANI/CNT-Based Sensor

**Fig. 4:**
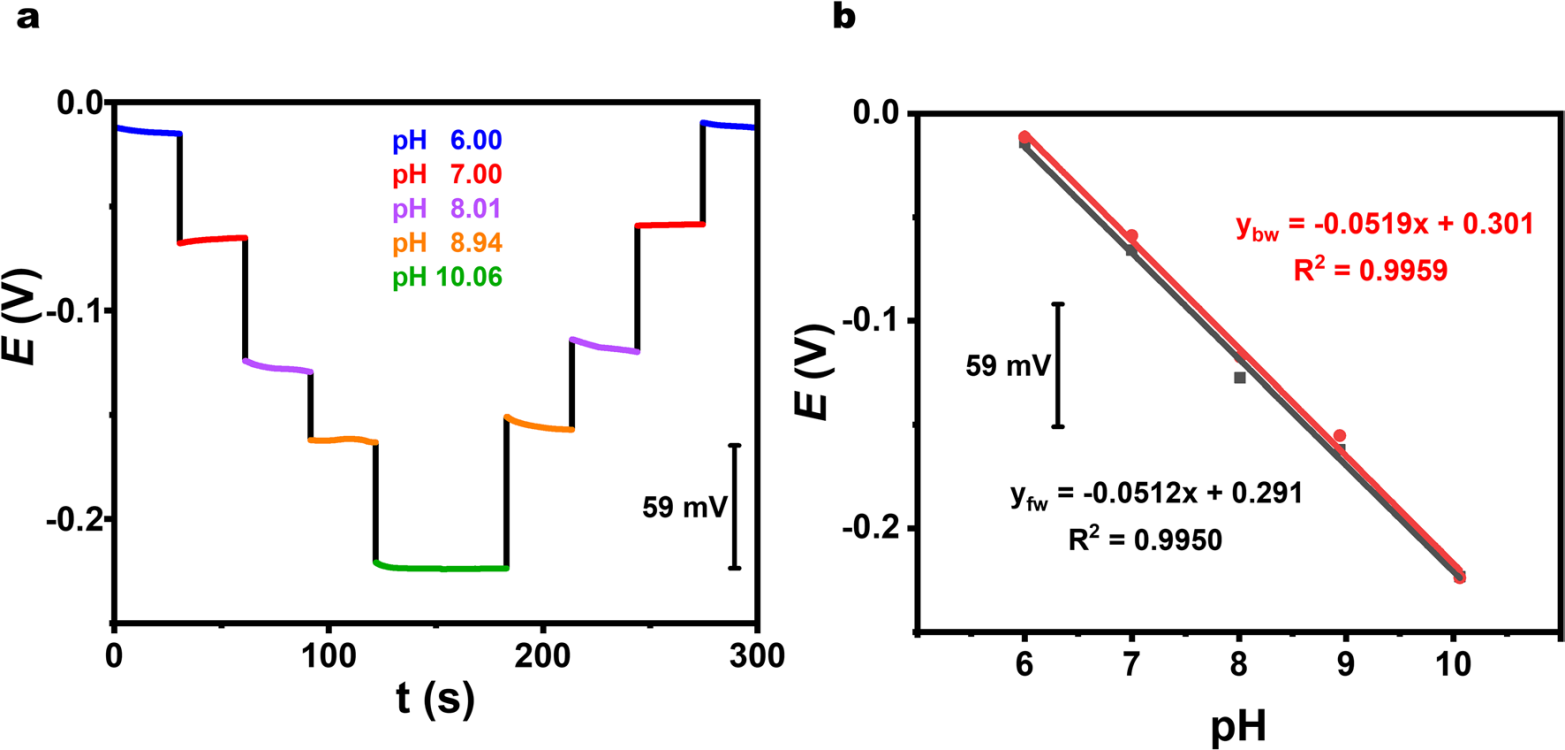
Investigation of potentiometric responses of the bandage-based pH sensor. **a** Potential-time response of the potentiometric pH sensor to various pH solutions. **b** Calibration plot depicting pH versus potential output, including linear fits for forward and backward responses

Our bandage was integrated with a nanocomposite film tailored to be sensitive within the typical pH range of human wounds (pH 6.5–8.5) [45], crucial for infection detection and healing monitoring, influenced by microbial growth and enzyme activity [25]. Even though pH can vary significantly, it can provide additional support for obtaining better and more comprehensive information about wounds. Simultaneously, pH-dependent electroactive species reflect changes in the redox state of the wound environment, as these species undergo oxidation or reduction at varying pH levels. This can indicate the presence of specific metabolites, such as those produced by bacteria during infection, as well as markers of inflammation or healing progression. Relying solely on pH may not provide a comprehensive enough assessment to reliably predict wound infection. However, it is still important to recognize that pH serves as a significant indicator and can offer supportive insights into wound infection [46]. Fluctuations in pH within wound fluids can introduce deviations in other biosensors. Consequently, accurately detecting other metabolites necessitates a thorough understanding of pH dynamics. Therefore, it is essential to monitor pH levels diligently and implement appropriate correction systems. Effective monitoring in dynamic wound exudate demands sensors with minimal carryover. To assess this, we tested various pH solutions without reconditioning the bandage surface. Sequential tests exposed it to pH solutions, transitioning from low to high pH and then reversing the order. Figure 4a shows the output voltage of our flexible potentiometric sensor across pH levels from 6.0 to 10.0 and back. Figure 4b depicts the plot of pH sensor response against the pH of the buffer solution. Our pH sensor demonstrated a sensitivity of − 51.9 mV pH^–1^, accompanied by a coefficient of determination (*R*^2^) of 0.9962. Consistency in voltage output during transitions between pH levels in both directions indicates negligible carryover. The sensor’s high sensitivity, attributed to the polyaniline-based nanocomposite material, arises from its high conductivity and chemical structure [47], facilitating reversible protonation and deprotonation reactions in response to pH changes [48]. The response time to the change in the pH solution was fast due to the presence of polyaniline in the nanocomposite film. Polyaniline is a conducting conjugated polymer derived from the aniline monomer. Its polymeric chain contains amide and imide groups, which enable reversible protonation and deprotonation [24, 49, 50]. This reversible protonation/deprotonation process results in a change in potential. For instance, when the pH increases, deprotonation occurs, leading to a decrease in potential. Conversely, if the pH increases, the number of protons increases, causing protonation and subsequently an increase in potential. Moreover, the nanocomposite’s high surface area and porous structure enhance interaction with the fluid, enabling rapid and sensitive pH detection [51].

### Investigating the Cross-Talk Effect of Wearable pH Sensors

When evaluating the performance of the bandage-based pH sensor, it is crucial to check potential cross-talk effects arising from varying pyocyanin concentrations to ensure reliable applicability across real scenarios. The selectivity test of the bandage-based wound sensor was conducted, starting with a fixed pyocyanin concentration, while recording voltage data across a pH range of 5.0 to 9.0. Results revealed that the PANI/CNT film of the printed bandage-based pH electrode exhibited clear responses to pH variations from 6.0 to 10.0, as evidenced by the distinct sensitive pattern (observed in Fig. S10, red plot). The slope from the corresponding calibration plot was found to be − 139 mV pH^−1^. Furthermore, in the second set of experiments, we varied pyocyanin concentrations from 1 × 10^−10^ to 1 × 10^−3^ M within a fixed pH of 8.0. Remarkably, changing pyocyanin concentrations across this wide range resulted in negligible changes in potential output (as depicted by the black plot in Fig. S10). These findings underscore the reliability of the bandage-based pH sensor, as the PANI/CNT film of the printed bandage-based pH electrode selectively responds solely to pH changes while minimizing cross-interference from pyocyanin, thereby ensuring accurate measurements in wound exudate environments.

We have further conducted a comprehensive investigation into the interference effect on the pseudoreference electrode. In this study, a pseudoreference electrode is prepared using a reference cocktail that contains polyvinyl butyral (PVB) as one of its key components. A robust reference electrode employing PVB demonstrates consistent potential stability [52]. To evaluate the functionality of the pseudoreference electrode, we measured the open-circuit voltage (OCV) between the pseudoreference electrode with PVB-NaCl and the standard reference electrode (Ag/AgCl electrode with 3.0 M KCl internal solution). Additionally, we introduced potential interferences (such as creatine, uric acid, creatinine, ascorbic acid, glucose, sodium bicarbonate, and lactate) into different solutions to assess the ionic selectivity of the pseudoreference electrode. The concentrations of interfering agents ranged from 1 to 10 times higher than those documented in the wound literature [42, 43, 53, 54]. As shown in Fig. S11, there were no significant changes in OCV readings (pseudoreference electrode with PVB-NaCl vs. Ag/AgCl) across varying concentrations of interfering agents. These results underscore the accuracy of the pseudoreference electrode in maintaining consistent potentiometric measurements, even in the presence of potential interfering substances.

### Development of Detection Model with pH-Correction System

**Fig. 5:**
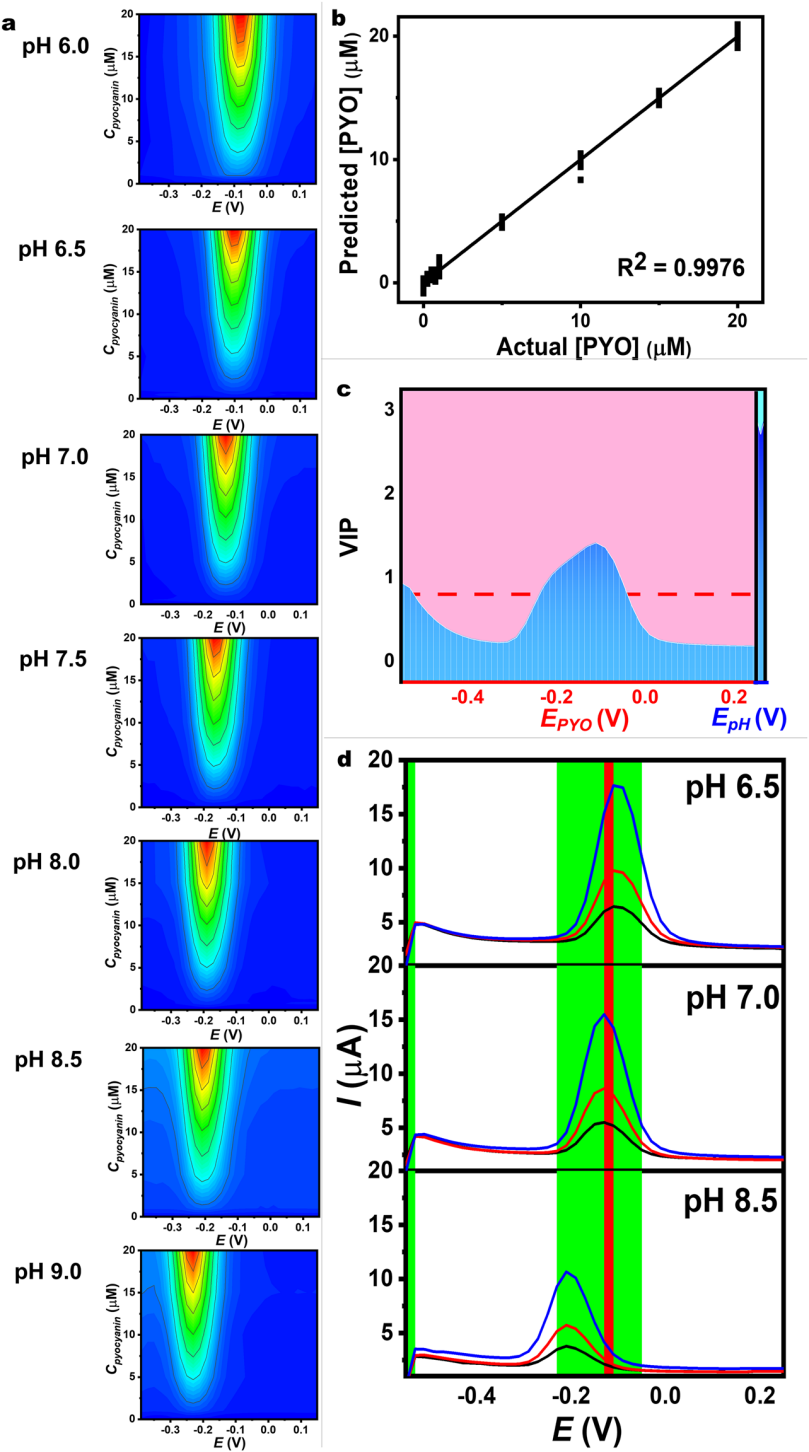
Development of the detection model with pH correction. **a** Color contour profiles showing current responses at different applied potentials and pyocyanin concentrations. Signals were obtained after background subtraction. **b** PLS regression curve to estimate the pyocyanin concentration using full voltammetric data over the applied potential range of − 0.55 to 0.25 V versus Ag/AgCl together with pH potentiometric data. **c** VIP values from the model. The data from the pyocyanin sensor using SWVs are shown in pink shade, and the data from the pH sensor using potentiometry are shown in blue shade. **d** Voltammetric outputs obtained for (1) 5.00 μM (black line), (2) 10.00 μM (red line), and (3) 20.00 μM (blue line) pyocyanin in pH 6.5, 7.0, and 8.5. The shaded green area and red area emphasize the range of important applied potentials where the VIP value exceeds 0.8 and the highest first-quartile VIP values

On the porous CNT/graphene pyocyanin sensor, we conducted measurements of pyocyanin concentrations in various fluids with different pH values. In Figs. 5a and S12, the results of the current outputs are presented, with two variables controlled: applied potential and levels of pyocyanin in different pH solutions. The corresponding mapping plot provides a graphical representation to illustrate the relationship between all three variables on a 2-dimensional plane of different applied potentials and pyocyanin concentrations. This visualization aids in understanding how changes in independent variables (i.e., applied potentials and pyocyanin concentrations) affect the observed current responses (i.e., dependent variable). The SWVs and corresponding contour plots demonstrate that the current responses of the well-defined SWVs are influenced by pyocyanin concentration across all pH conditions. The contour plot corresponding to the SWVs indicates the optimal applied potential for detecting pyocyanin at each pH condition. The optimal potential was determined by the location that yields clear differences in current output.

Numerous multivariate regression techniques exist for predicting outcomes using multiple independent variables, such as multiple linear regression. However, these methods often encounter challenges when dealing with datasets characterized by an abundance of predictors. Partial Least Squares (PLS) regression emerges as a solution to address these concerns by effectively reducing the dataset's dimensionality. Unlike merely discovering a single parameter, PLS regression utilizes a collection of internally constructed parameters within the model [55]. The proficiency of the PLS algorithm in managing complex datasets highlights its essential role in analytical chemistry for real sample analysis [56], such as constructing a predictive model for amylose content in rice [57], and estimating the concentration of different components in wine [58]. In this work, PLS regression method was utilized to construct our analytical model, establishing a relationship between numerous independent variables and response variables. PLS regression is advantageous for complex on-body analytical chemistry as it incorporates full available data on current outputs, applied potentials, pyocyanin concentration, and pH to build a calibration model. This model predicts unknown values of dependent variables, namely pyocyanin concentration and pH. To construct the calibration model, we utilized an experimental dataset. For each experimental test, we gathered 44 data features, including 41 current data points from SWV at various potentials and 3 pH data points from pH potentiometric detection. Integrating voltage values (corresponding to pH fluctuations) obtained from the pH sensor with current outputs from the pyocyanin sensor resulted in our proposed calibration model, aiming for greater robustness compared to the traditional linear calibration model.

Figure 5b illustrates the model corresponding to different concentrations of pyocyanin. The obtained model accurately predicts pyocyanin concentration, as evidenced by its high coefficient of determination (*R*^2^ = 0.9957). The calibration curve of the PLS model for pH value prediction also demonstrates an *R*^2^ of 0.9961 (Fig. S13), indicating the model’s acceptable prediction performance.

The variable importance of projection (VIP) value was considered to assess the significance of various applied potentials to the resulting signals determining pyocyanin. Figure 5c displays the VIP values from PLS analysis for potential variables from the pyocyanin sensor and pH sensor, indicating the significance of each applied potential (from the pyocyanin sensor) and pH value (from the pH sensor) for pyocyanin quantification. SWV curves highlighted in red represent voltammetric variables with the highest first-quartile-rank VIP values, while the green color indicates VIP values exceeding the cutoff of 0.8 (Fig. 5d). In the detection of pyocyanin, applied voltages within the critical range of − 0.23 to − 0.05 V (highlighted in green) correspond to a specific range of − 0.25 to − 0.07 V, which aligns with the oxidation potentials observed across a wide variation in pH wound matrix media, around pH 6.0 to 9.0.

### Mechanical Resilience of Printed Bandage-Based Sensors

**Fig. 6:**
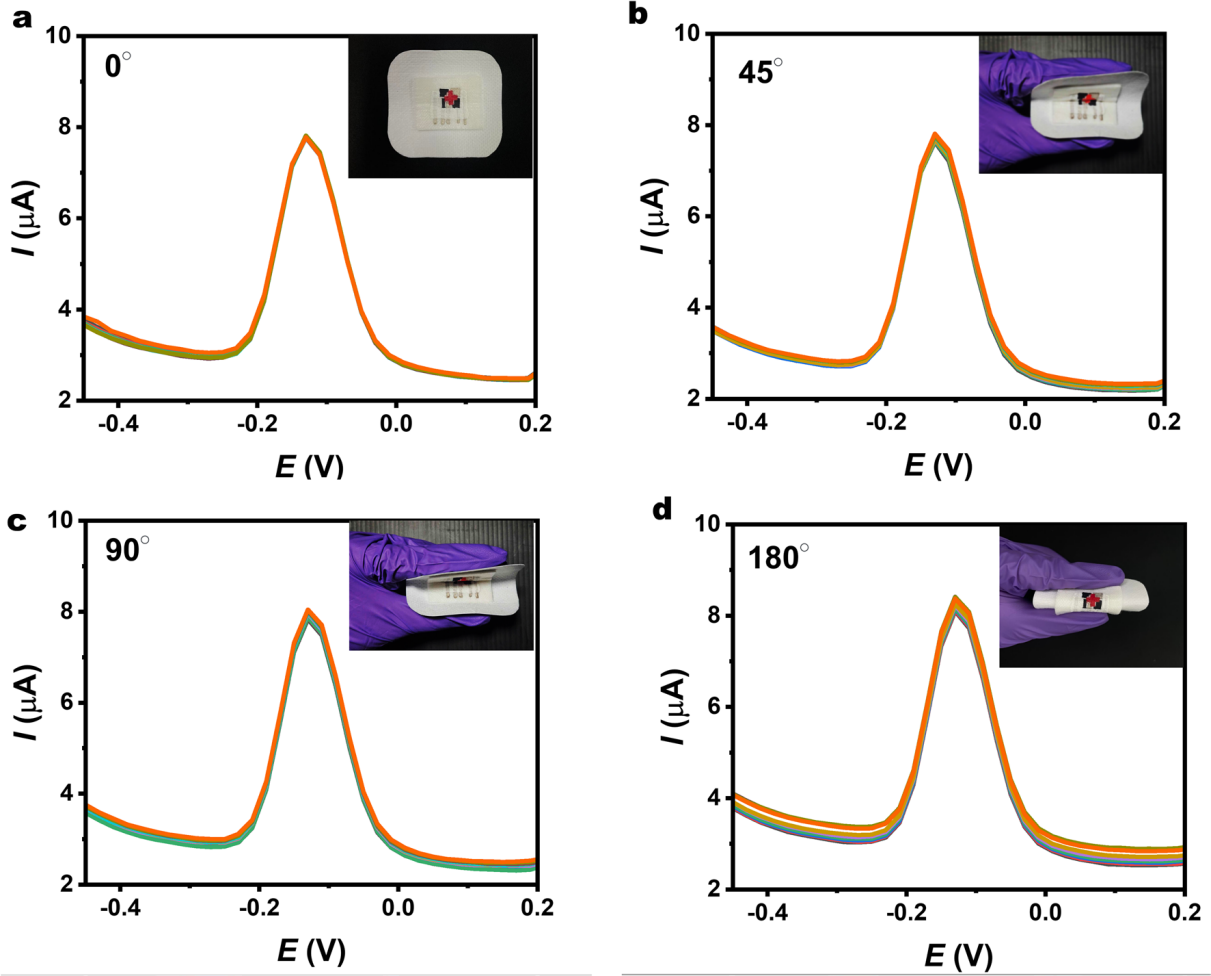
SWV curves of the porous CNT/graphene electrode were recorded during a series of 100 bending iterations, with each iteration involving 10 repetitions, under varying mechanical bending angles **a** 0°, **b** 45°, **c** 90°, and **d** 180° in 10 µM pyocyanin

To evaluate potential mechanical distortion effects on the electrochemical performance of the printed material when worn as a bandage, replicating the bending scenario is crucial. Thus, the porous CNT/graphene electrode was bent into various angles to investigate the mechanical resilience of the printed device (Fig. 6). The flexible device demonstrated favorable mechanical stability when bent at different angles, with an overlap of SWV curves observed at bending angles of 0°, 45°, 90°, and 180°, indicating the device's electrochemical stability was maintained. A durability test, consisting of 400 cycles under curved conditions, was conducted, suggesting that the electrode possesses the desired functional characteristics. Importantly, this indicates that our screen-printable inks offer highly stable performance and mechanical resilience. The absence of severe impacts from such repeated distortion ensures the device maintains optimal functionality and reliability during real-world usage.

### Use of Printed Bandage-Based Electrochemical Sensing Array with pH-Correction Model in Complex Matrix

**Fig. 7:**
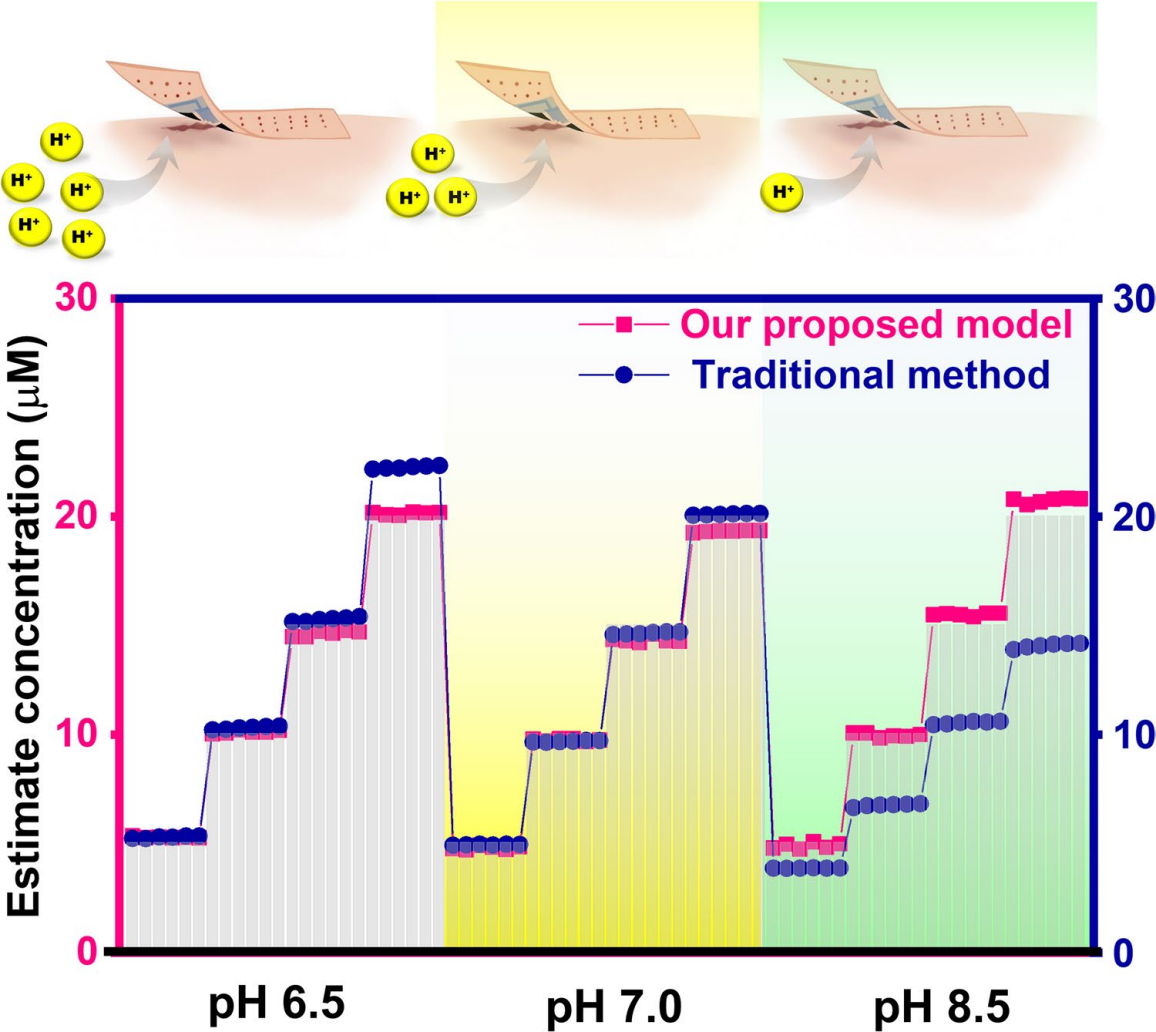
Comparison of estimated pyocyanin concentrations obtained from our proposed pH-correction model and traditional calibration method at various pH levels of simulated wound fluids. Bar charts depict standard control concentrations of pyocyanin available in the fluids

To validate our pH-correction system, we tested modeled wound fluids at various concentrations of pyocyanin and pH levels, comparing them with the traditional system without pH-correction integration (Fig. 7). In the acidic region (pH 6.5), the traditional model significantly overestimates the concentration, while in the basic region (pH 8.5), it underestimates it. These discrepancies highlight the critical issue of neglecting pH fluctuations in dynamic on-body scenarios. Advantageously, our printed wearable bandage-based electrochemical sensing array, coupled with the correction model, can dynamically mitigate such errors. Using our proposed method, the predicted pyocyanin concentration closely matches the actual concentration across a wide range of pH values in biological samples [24, 25].

It is also crucial to consider the electrode's temporal response and its suitability for monitoring variations in pyocyanin concentration in wound environments, particularly in real-world scenarios such as the decrease in pyocyanin levels due to antibiotic treatment or its increase. In this section, continuous experimental setups were conducted using the same electrode (Fig. 7). The results demonstrate the precision of our newly developed pyocyanin sensor when combined with the proposed pH-correction model, validated by comparing the results with known pyocyanin concentrations. This validation confirms that our sensor exhibits no significant issues regarding pyocyanin adhesion on the electrode surface at various pH settings relevant to possible stages of the infection progression.

To assess the analytical performance of different calibration models for estimating pyocyanin concentration, recovery analysis was conducted on various pyocyanin concentrations and pH levels. When the pH of the fluids shifts from neutral to acidic or basic, our system utilizing a printed bandage-based sensing array demonstrates greater accuracy, yielding acceptable recovery values compared to the traditional model, as shown in Table S3. Under controlled conditions at neutral pH, traditional benchtop analysis yields satisfactory results (recovery ranging from 94.43 ± 0.02% to 100.79 ± 0.02%). However, implementing this traditional approach in on-body settings presents significant challenges due to the manipulation of small volumes of biofluids and the complexity of sample pretreatment procedures on the small wearable bandage. Traditional calibration methods, when applied without the integrated PANI/CNT sensor for correction, exhibit considerable errors in acidic and basic conditions. These errors are due to the direct influence of pH on the material and electrochemical behavior of pyocyanin oxidation, resulting in recoveries exceeding 123% in acidic conditions and dropping below 35% in basic conditions. In contrast, the proposed model, leveraging a printed wearable bandage-based electrochemical sensing array along with the correction model, demonstrates high performance. The recovery for the proposed model falls within the range of 93.55 ± 0.01% to 106.75 ± 0.12%, which is closer to the desired 100% recovery. This indicates that the proposed system maintains precision even in the face of fluctuating pH levels. The integration of the correction model addresses the challenges posed by varying pH conditions, thereby enhancing the method’s adaptability in real-world applications. By mitigating the influence of pH fluctuations on the material and electrochemical behavior of pyocyanin, the proposed system offers a practical solution for on-body monitoring, where reliable detection is crucial.

### Integration of Electrochemical Sensor with Wireless Portable Instrument for Monitoring Bacterial Growth

The concept of the proposed bandage setup, as illustrated in Fig. S14, involves sensors printed directly onto the bandage, allowing for the measurement of biomarkers in wound fluid and real-time data transmission to users via a wireless device. Specifically, the system utilizes fully-printed porous graphene/CNT and PANI/CNT composites as pyocyanin and pH sensors. These sensors are integrated with a compact wireless device that records current and voltage outputs, transmitting the data for immediate analysis.

Recognizing the importance of monitoring pyocyanin in real bacterial growth environments, we evaluated the feasibility of using our sensors integrated with a wireless portable instrument. This study aimed to investigate the relationship between the initial bacterial cell counts of *P. aeruginosa*, their subsequent growth, and pyocyanin production. The initial bacterial inoculates were set at 10^1^, 10^3^, and 10^5^ CFU mL^–1^.

We utilized the porous CNT/graphene electrode connected to a wireless electronic device to measure the increase in pyocyanin secretion from bacterial cultures, observing the current response (Fig. S15a). We observed that the oxidation peak current of pyocyanin increased proportionally with initial bacterial counts, indicating bacterial growth in the simulated wound setup. This trend highlights a direct correlation between initial bacterial load and pyocyanin production. In parallel, spectrophotometry was employed to further validate pyocyanin production. We measured optical density at 520 nm for pyocyanin pigment production and at 600 nm to assess bacterial growth (Fig. S15b and c). Additionally, viable cell counts were performed throughout the experiment (Fig. S15d). These spectrophotometric analyses, coupled with observations of cell growth, complemented the electrochemical data, providing additional confirmation of pyocyanin production and enhancing the potential for using pyocyanin as a biomarker for bacterial infection warning systems.

Biocompatibility is a key factor in selecting materials. We prioritized materials with proven compatibility for noninvasive, on-skin use. To evaluate biocompatibility, we conducted tests using L-929 mouse fibroblast cells, a standard model for assessing cellular responses. As shown in Fig. S16, the sensor materials exhibited high cell survival rates, confirming their non-toxicity and suitability for biomedical applications.

## Conclusions

This work describes a flexible printed sensing array integrated onto a bandage surface, capable of monitoring both pyocyanin and pH levels in wound exudate. This presents an advantageous strategy for interpreting analytical signals amidst pH variations in complex biofluids. Fabrication of the sensitive pyocyanin and pH sensors involved the use of printed porous CNT/graphene-based materials to facilitate the electrooxidation of the metabolite indicating bacterial presence, and the creation of a flexible pH-sensitive membrane using PANI/CNT-based materials. The sensing array, prepared with customized screen-printable inks, is simple, affordable, sensitive, and enables rapid detection. Electrochemical and physical analyses, including contact angle measurements, and SEM/EDX imaging, were employed to assess electrode properties. Comparison between unmodified and porous CNT/graphene electrodes revealed that the porous CNT/graphene electrode exhibited superior effectiveness. The improvement was due to its porous nature and material composites, providing a large active surface area for interaction with the solution. To address the challenge of pH dynamic fluctuations when measuring pH-dependent electroactive species, we developed a mathematical pH-correction system based on the PLS model. This system utilizes potentiometric data from the pH sensor and voltammetric current data from the pyocyanin sensor. Consequently, the accuracy of pyocyanin determination was enhanced. This demonstration has the potential to expedite the identification and treatment of wound infections by integrating multiple complex sensors into wearable devices, thereby overcoming obstacles associated with monitoring wounds in biofluids with high pH variation. Importantly, this strategic concept serves as a foundational model for advancing other flexible, on-body, or on-demand sensors. The idea of this convenient flexible electrochemical sensing array and the integrated correction system can better meet the requirements of various modern analytical chemistry fields, where resilience to mechanical deformations and simplicity of analysis are essential.

## Supplementary Information

Below is the link to the electronic supplementary material.

Supplementary file1 (PDF 982 KB)
