# Supplementary material for: A Fully-Printed Wearable Bandage-Based Electrochemical Sensor with pH Correction for Wound Infection Monitoring
Source: Nanomicro Lett. 2024 Nov 26;17:71. doi: 10.1007/s40820-024-01561-8 (PMC11599554; doi:10.1007/s40820-024-01561-8)
Supplement: Supplementary file 1 — Supplementary file1 (PDF 982 KB) [file 40820_2024_1561_MOESM1_ESM.pdf]

Supporting Information

A fully-printed wearable bandage-based electrochemical sensor with pH correction for wound infection monitoring.

Kanyawee Kaewpradub[1,2,4]*, Kornautchaya Veenuttranon*[1,4]*, Husanai Jantapaso*[5]*,*
Pimonsri Mittraparp-arthorn[5]*, Itthipon Jeerapan*[1,2,3,4*]

[1] *Center of Excellence for Trace Analysis and Biosensor, Prince of Songkla University, Hat Yai, Songkhla 90110, Thailand*

[2] *Division of Physical Science, Faculty of Science, Prince of Songkla University, Hat Yai, Songkhla 90110, Thailand*

[3] *Center of Excellence for Innovation in Chemistry, Faculty of Science, Prince of Songkla University, Hat Yai, Songkhla 90110, Thailand*

[4] *The ijE Electrochemistry for All Laboratory, Prince of Songkla University, Hat Yai, Songkhla 90110, Thailand*

[5] *Division of Biological Science, Faculty of Science, Prince of Songkla University, Hat Yai, Songkhla, 90110, Thailand*

[*]*Corresponding author: e-mail address: itthipon.j@psu.ac.th (Itthipon Jeerapan)*
ORCID of Itthipon Jeerapan: https://orcid.org/0000-0001-8016-6411 (Itthipon Jeerapan)

Supporting Experimental Section

1. Chemicals and reagents

Pyocyanin, aniline, potassium hexacyanoferrate (III) ($K_3[Fe(CN)_6]$), L-ascorbic acid, uric acid, lactic acid, creatine monohydrate, polyvinyl butyral (PVB), sodium deoxycholate, sodium dodecyl sulfate, and creatinine were from Sigma Aldrich. Glucose and urea were from Fluka (USA). Sodium chloride (NaCl), sodium bicarbonate ($NaHCO_3$), calcium chloride dihydrate ($CaCl_2{\cdot}2H_2O$), and calcium carbonate ($CaCO_3$) were from Merck (Germany). Magnesium chloride ($MgCl_2{\cdot}6H_2O$) was from Carlo Erba Reagents (Germany). Potassium hexacyanoferrate (II) trihydrate ($K_4Fe(CN)_6{\cdot}\ 3H_2O$) was brought from RDH Laborchemikalien GmbH & Co. KG., Germany. Sodium carbonate (Na_2CO_3) was from Ajax Finechem. Hydrochloric acid (HCl) and methanol were from RCI Labscan Limited (Ireland). Sulfuric acid (H_2SO_4) was from Labscan Asia Co., Ltd. (Thailand), Toluene was from Guangdong Guanghua Chemical Factory Co., Ltd. (China), Multiwalled carbon nanotubes (MWCNTs, diameter: 60–100 nm, length: >5 μm, special surface area: 40–70 $m^2\ g^{-1}$) was from Shenzhen Nanotech Port Co., Ltd. (China). Graphene conductive ink (UGDC033SSCDSV) was from UGENT company (Malaysia). Carbon conductive ink (DSNSMF0001) was from DSN thermal solution (China). Ag/AgCl ink was from Sun Chemical Ltd. (UK). Ecoflex® 50 was from Smooth-On, Inc. (USA). Tetrahydrofuran (THF) was purchased from Honeywell, B&J brand, USA. All reagents were used without further purification. All chemical solutions were prepared using ultrapure water (18.2 MΩ cm) from a Milli Q Merck system (Germany). Tryptic Soy Agar (TSA) and Mueller Hinton broth (MHB) were purchased from Difco, Becton, Dickinson and company (USA).

2. Preparation of artificial wound

Artificial wound used in this experiment to test the ability of the sensor is the modified form [1], prepared by mixing 1.00×10^{-3} M of glucose, 9.00×10^{-4} M of urea, 5.60×10^{-3} M of $CaCl_2$, 8.00×10^{-8} M of $MgCl_2$, 1.9×10^{-4} M of NaCl, and 1.00×10^{-5} M of $NaHCO_3$.

3. Preparation of MHB broth (1X)

21 g of MHB powder was suspended in 1 L of water and gently warmed to dissolve. Then, it was autoclaved at 121 °C for 15 min.

4. Preparation of TSA agar (1X)

40 g of TSA powder was suspended in 1 L of water and gently warmed to dissolve. Then, it was autoclaved at 121 °C for 15 min.

5. Biocompatibility test using MTT assay

The biocompatibility of the sensor materials was assessed using L-929 cells through the 3-(4,5-dimethylthiazol-2-yl)-2,5-diphenyltetrazolium bromide (MTT) assay. Specifically, L-929 cells (Earles's cells, L cells, or NCTC clone 929 cells from fibroblast tissues) were obtained from ATCC (Manassas, VA, USA) and cultured in a 1:1 mixture of Dulbecco's Modified Eagle Medium (D-MEM), supplemented with 10% fetal bovine serum (FBS), 100 U mL^{-1} penicillin, and 100 μg mL^{-1} streptomycin. The cultures were maintained in a humidified incubator at 37 °C with 5% CO_2 and incubated for 72 hours. For the cytotoxicity assay, the sensor was exposed to UV light for 30 min, then soaked in cell culture medium for 5 hours. Afterward, the electrodes were removed, and L-929 cells were seeded into 96-well plates at a density of 8,000 cells per well. The electrode-soaked medium was added to the wells in a 1:1 ratio with fresh culture medium, and the plates were incubated at 37 °C for 72 hours. After incubation, the cells were washed with 1X PBS (pH 7.4) and incubated with 100 µL of 0.5 mg mL^{-1} MTT at 37 °C with 5% CO_2 for 30 min. Dark blue formazan crystals, indicative of MTT metabolism, were then dissolved in 100 µL of dimethyl sulfoxide (DMSO) at room temperature, protected from light. The plates were incubated again at 37 °C for 30 min in the dark and absorbance was measured using a microplate reader spectrophotometer at 570 and 650 nm. Percent survival was calculated using the formula:

$$\text{Survival (\%)} = \frac{(A_{sample,570} - A_{sample,650})}{(A_{control,570} - A_{control,650})} \times 100$$

where $A_{sample,570}$, $A_{sample,650}$, $A_{control,570}$, and $A_{control,650}$ are the absorbance of the test samples (at 570 and 650 nm) and negative control (at 570 and 650 nm), respectively [2].

6. Instrumentation

Electrochemical experiments were conducted using a µAutolab II potentiostat (Metrohm Autolab, The Netherlands) and controlled by the Nova 2.1 software to investigate the electrochemical performances via cyclic voltammetry (CV), square wave voltammetry (SWV), and electrochemical impedance spectroscopy (EIS). The three-electrode system used in SWV and EIS experiments. SWV experiments consisted of a flexible printed working electrode (e.g., the porous CNT/graphene electrode), a carbon counter, and a Ag/AgCl pseudoreference electrode. Square wave voltammetry (SWV) was chosen for pyocyanin

analysis. The SWV detection was carried out at a frequency of 10 Hz, with a step potential of 20 mV, and an amplitude of 50 mV. Each set of experiments was performed with three replicates (n = 3). The EIS experiments used a root mean square (RMS) amplitude of 10 mV, employing a single sine wave type. The bias potential was set at +0.60 V for the unmodified electrode and at +0.35 V for the porous CNT/graphene electrode. The frequency sweep spanned from 1×10^4 Hz down to 1×10^{-1} Hz. The two-electrode system used in potentiometric experiments consisted of a flexible working (PANI/CNT) and a Ag/AgCl pseudoreference electrode. A compact voltmeter (Vernier GO Direct® Electrode Amplifier) was used to record the output voltage of the pH sensor. Potentiometric measurement was carried out by recording the electrochemical potential of the two electrodes of pH sensor to perform pH detection. The contact angle was calculated using ImageJ 1.50a software package (National Institutes of Health, Bethesda, MD, USA). Characterize morphology of electrodes using SEM/EDX technique used field emission scanning electron microscope (FESEM) (Apreo model, Brand FEI, Netherlands). Bacterial growth and pyocyanin production were measured by optical density using a microplate reader (LUMIstar Omega, Germany).

Supplementary Figures

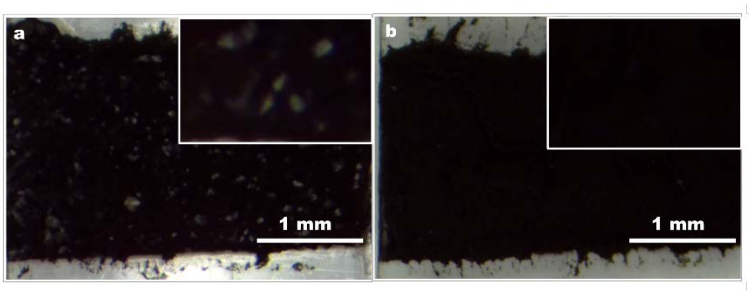

Fig. S1. Surface characterization by optical microscopy of **a** non-porous CNT/graphene electrodes (before $CaCO_3$ removal) and **b** porous CNT/graphene electrodes (after $CaCO_3$ removal). Insets depict images digitally magnified 5 times.

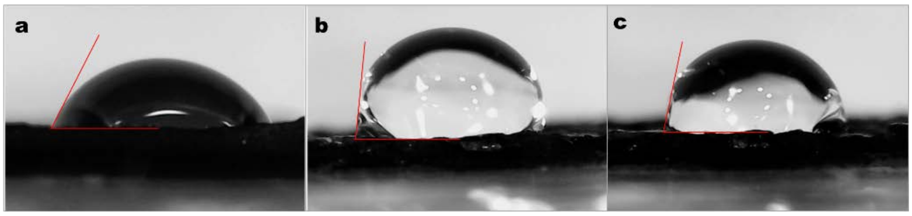

Fig. S2. Contact angle measurements of **a** unmodified ink electrode, **b** porous CNT/graphene electrode without electrochemical treatment, and **c** porous CNT/graphene electrode with electrochemical treatment via anodization in Na_2CO_3 solution.

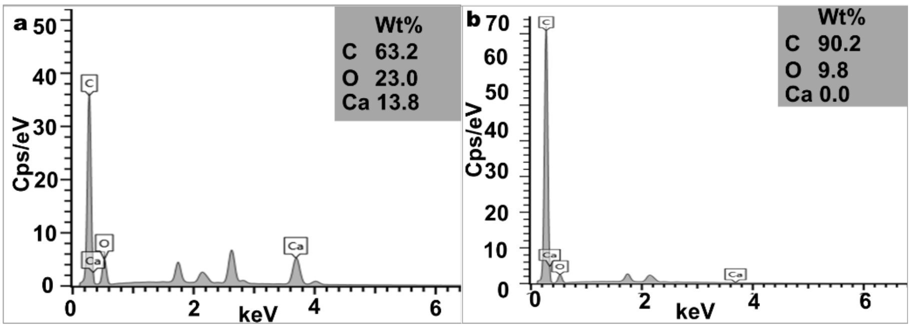

Fig. S3. The characterization using energy dispersive X-ray spectroscopy (EDX) spectrum analysis. **a** EDX spectrum of the CNT/graphene electrode (before etching to remove $CaCO_3$) and **b** the resulting porous CNT/graphene electrode (after $CaCO_3$ removal).

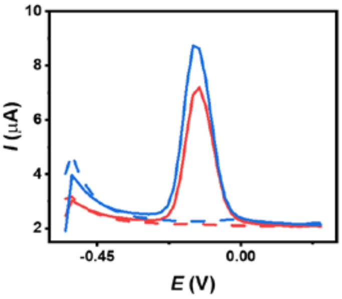

Fig. S4. SWVs obtained from a non-porous CNT/graphene electrode (red line) before removal of $CaCO_3$ and a porous CNT/graphene electrode (blue line) after $CaCO_3$ removal in the absence (dash line) and presence (solid line) of 10 µM pyocyanin in 0.10 M PBS, pH 7.00.

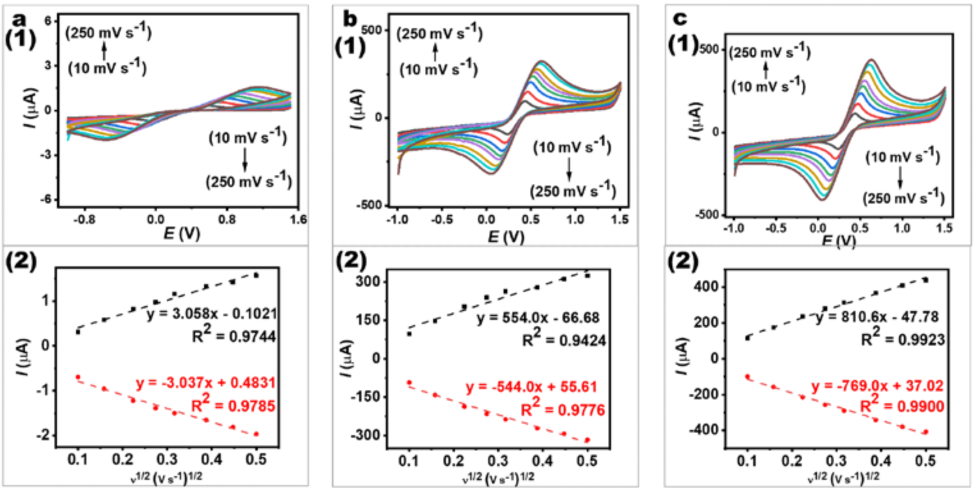

Fig. S5. Electrochemical performances of different printed electrodes: **a** unmodified material electrode, **b** the CNT/graphene electrode before $CaCO_3$ removal, and **c** porous CNT/graphene electrode after $CaCO_3$ removal. (1) The experiments were conducted using CV in the presence of 10 mM $K_3[Fe(CN)_6]$ in 1.0 M KCl at various scan rates ranging from 10 to 250 mV s^{-1}. (2) Anodic and cathodic peak current densities plotted against the square root of the scan rates.

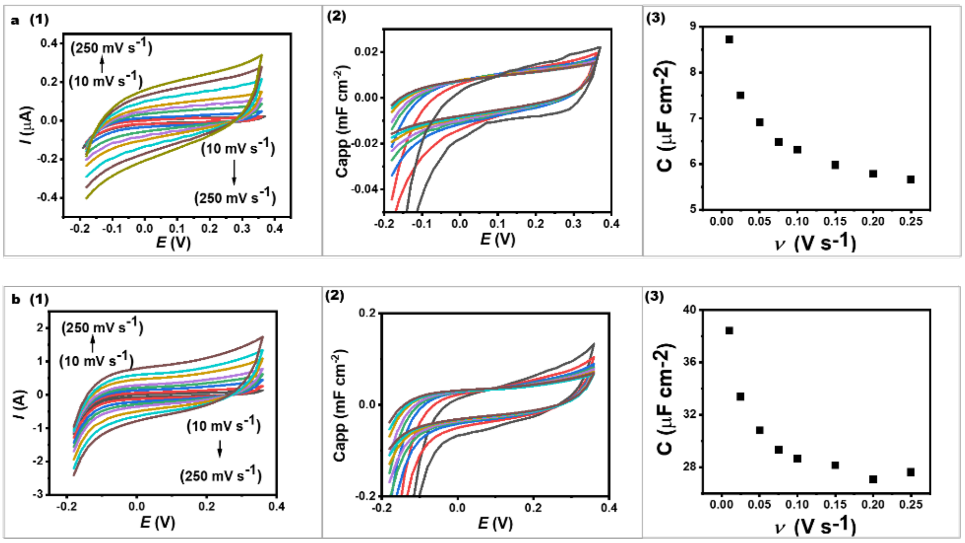

Fig. S6. Surface study of various printed electrodes in electrolytes, including **a** unmodified material electrode and **b** the porous CNT/graphene electrode after $CaCO_3$ removal. The investigations include: (1) Cyclic voltammograms (CVs) obtained in 1.0 M KCl at scan rates ranging from 10 to 250 mV s^{-1}. (2) Apparent capacitance profile. (3) Plot depicting capacitance variations at different scan rates.

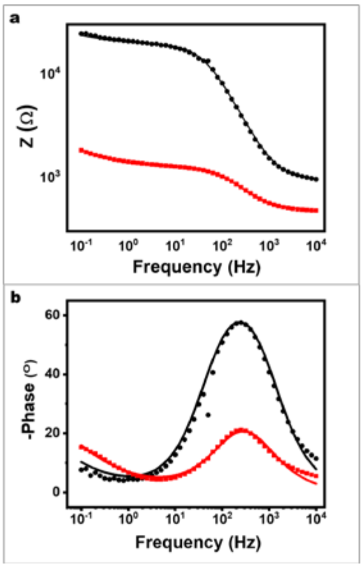

Fig. S7. Bode plots obtained from printed electrodes, including the unmodified material electrode (black plot) and the porous CNT/graphene electrode after $CaCO_3$ removal (red plot). Electrochemical impedance spectroscopy (EIS) studies were conducted in 2.5 mM $K_3[Fe(CN)_6]$ and 2.5 mM $K_4[Fe(CN)_6]$ in 1.0 M KCl, utilizing a frequency range of $1{\times}10^{-1}$–10^4 Hz, an amplitude of 10 mV, 0.60 V DC bias for unmodified electrode, and potential apply at 0.35 V DC bias for porous CNT/graphene electrode. The solid lines over the black dot or red square points represent the fitted graphs following the equivalent circuit model, corresponding to Fig 2c.

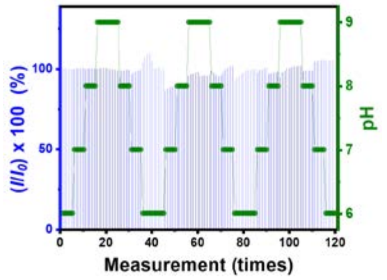

Fig. S8. Stability test of pyocyanin measurements across varying pH values. The pH was increased from 6.0 to 9.0, and also decreased from 9.0 to 6.0.

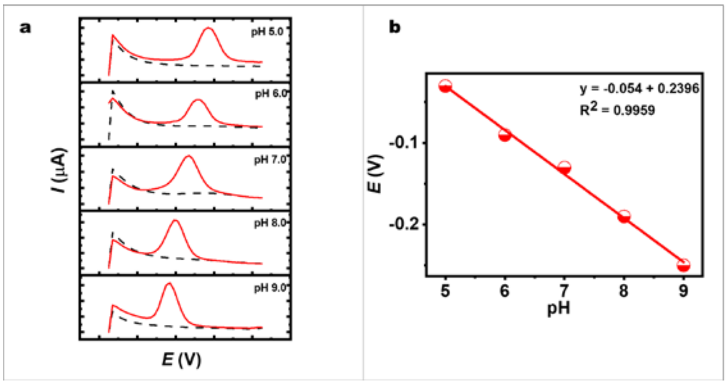

Fig. S9. a SWV illustrating the influence of pH on the electrochemical behavior of pyocyanin oxidation (with 10 μM pyocyanin across pH levels ranging from 5.0 to 9.0). **b** Corresponding relationship plot showing the pyocyanin oxidation peak position relative to medium solution pH.

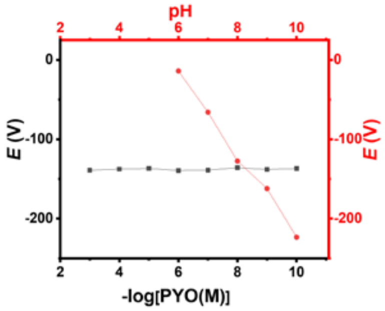

Fig. S10. Selectivity of the bandage-based pH sensor. **Set I (Red plot):** Pyocyanin concentration fixed at 10 μM. Voltage data recorded in solutions with pH ranging from 6.0 to 10.0. **Set II (Black plot)**: Pyocyanin concentration varied from 1×10^{-3} to 1×10^{-10} M, prepared in 0.1 M PBS at pH 8.0. Voltage data recorded for these concentrations at constant pH 8.0.

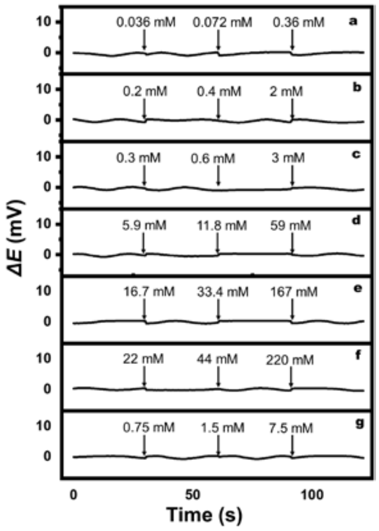

Fig. S11. Study the effect of possible interfering species on the reference electrode by varying the concentrations of additional species, including **a** ascorbic acid (0, 0.036, 0.072, and 0.36 mM), **b** creatine (0, 0.2, 0.4, and 2 mM), **c** creatinine (0, 0.3, 0.6, and 3 mM), **d** glucose (0, 5.9, 11.8, and 59 mM), **e** lactate (0, 16.7, 33.4, and 167 mM), **f** sodium bicarbonate (0, 22, 44, and 220 mM), and **g** uric acid (0, 0.75, 1.5, and 7.5 mM), added to an artificial wound solution. Changes in potential versus a standard reference Ag/AgCl electrode with a 3.0 M KCl internal solution were recorded.

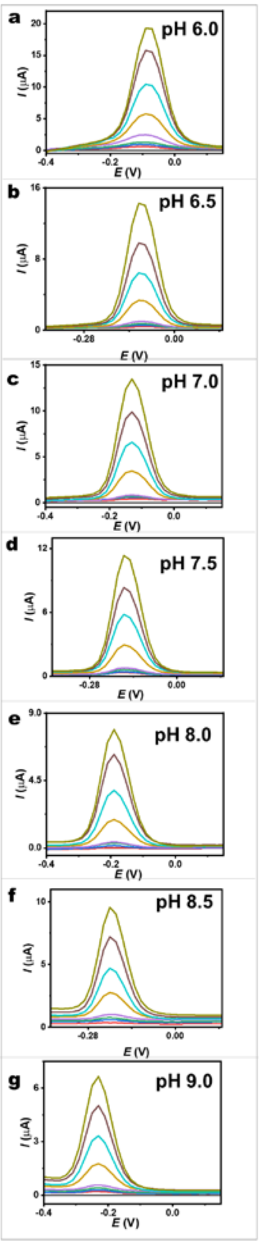

Fig. S12. Analytical signal dependence on pyocyanin concentration (0–20 µM) at various pH levels for pH **a** 6.0, **b** 6.5, **c** 7.0, **d** 7.5, **e** 8.0, **f** 8.5 and, **g** 9.0.

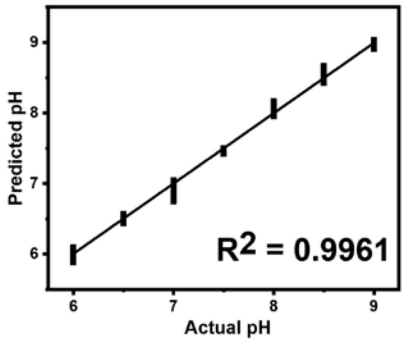

Fig. S13. PLS regression plot from the developed model for predicting pH levels.

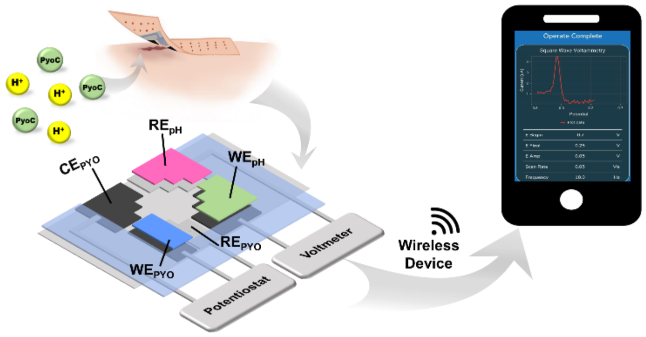

Fig. S14. Schematic illustration of a printed flexible sensing array for detecting pyocyanin and pH on a bandage, alongside the system's circuit configuration.

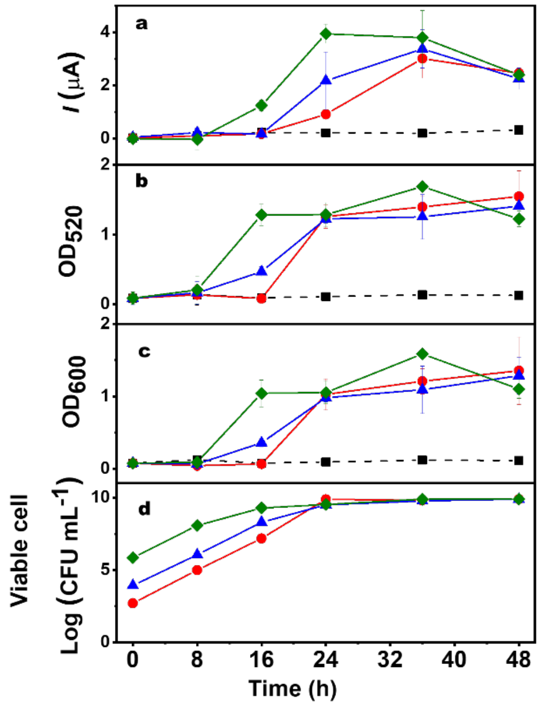

Fig. S15. a Current response, **b** optical density at 520 nm, **c** optical density at 600 nm, and **d** viable cell count in a simulated wound environment at three different initial bacterial inoculum levels including 0 (black line), 10^1 (red line), 10^3 (blue line), and 10^5 (green line) CFU mL^{-1}.

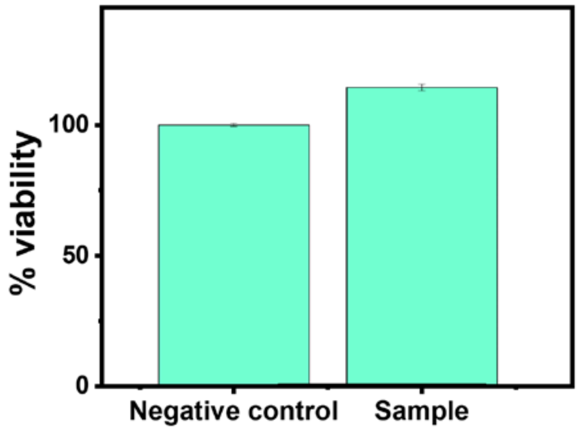

Fig. S16. Biocompatibility test for the materials in the printed wearable bandage sensor.

Supplementary Tables

Table S1 Electrochemical sensors using various materials for the detection of pyocyanin.

Electrodes	Electrochemical techniques	Integrating pH sensor	Printable	Wearable bandage	Range (µM)	LOD (µM)	Ref
agar Au-Ag nanoalloy/SPCE	SWV	✗	✗	✗	0.12 – 25	0.04	[3]
carbon fiber tow laminate working electrode	SWV	✗	✗	✗	1 – 100	0.03	[4]
carbon ink/photo paper	SWV	✗	✓	✗	1 – 40	0.10	[5]
carbon nanodots/disposable paper electrodes	DPV	✗	✓	✗	4.45 – 52.47	0.15	[6]
CNT/PVA hydrogel/SPCE	SWV	✗	✓	✗	100	0.48	[7]
dielectric paste /carbon graphite ink	SWV	✗	✓	✗	1-10	0.15	[8]
Au electrode	CV	✗	✗	✗	2 - 100	2.00	[9]
AuNPs/rGO/SPCE	DPV	✗	✗	✗	0-100	0.27	[10]
Au working electrode	SWV	✗	✗	✗	1-100	0.60	[11]
MIP/chitosan/AuNP/SPCE	CV and SWV	✗	✗	✗	1-100	0.74	[12]
PDMS/AuNPs/Au electrodes/glass substrate	DPV	✗	✗	✗	0.5-250	010	[13]
planar transparent macroelectrodes	SWV	✗	✗	✗	0.75-25	0.75	[14]
PMMA/Al_2O_3/PSSs/PPF/PET substrate	SWV	✗	✗	✗	1-250	1.00	[1]
Polyaniline/AuNPs /ITO electrodes	CV	✗	✗	✗	1.9-238	0.50	[15]
thin film BDD electrode	DPV	✗	✗	✗	5-50	2.06	[16]

Electrodes	Electrochemical techniques	Integrating pH sensor	Printable	Wearable bandage	Range (μM)	LOD (μM)	Ref
transparent carbon ultramicroelectrode arrays	SWV	✗	✗	✗	1-250	1.00	[17]
CoTAPc-MXene/GCE	DPV	✗	✗	✗	0.1-200	0.039	[18]
Printed porous CNT/graphene electrode	SWV	✓	✓	✓	0.25-100	0.22	Present work

Abbreviations: **Materials**: *Agar Au-Ag nanoalloy/SPCE* Agar gold-silver nanoalloy/screen printed carbon electrodes, *CNT/PVA hydrogel/SPCE* Carbon nanotube/Poly(vinyl alcohol) hydrogels/screen printed carbon electrodes, *Au working electrode* Gold working electrode, *AuNPs/rGO/SPCE* Gold nanoparticles/reduced graphene oxide/screen printed carbon electrodes, *MIP/Chitosan/AuNP/SPCE* Molecularly imprinted polymers/chitosan/gold nanoparticles/screen printed carbon electrodes, *PDMS/AuNPs/Au electrodes/glass substrate* Polydimethylsiloxane/gold nanoparticles/gold electrode/glass substrate, *PMMA/Al_2O_3/PSSs/PPF/PET substrate* Poly(methyl methacrylate)/aluminum oxide/polystyrene spheres/pyrolyzed photoresist film/poly(ethylene terephthalate) substrate, *Polyaniline/AuNPs/ITO electrodes* Polyaniline/gold nanoparticles/indium tin oxide electrodes, *thin film BDD electrode* Thin film boron-doped diamond electrode. *CoTAPc-MXene/GCE* cobalt(II) tetra-aminophthalocyanine and MXene composites/glassy carbon electrode. **Techniques**: *DPV* Differential pulse voltammetry, *SWV* Square wave voltammetry, *CV* Cyclic voltammetry.

Table S2 The EIS results showing parameter values obtained from equivalent circuit settings using different printed electrodes, including the unmodified material electrode and the porous CNT/graphene electrode after $CaCO_3$ removal.

Materials	Elements				
	R_s (Ω)	R_{ct} (kΩ)	CPE_{dl} Y_0 (S . s^N) (μMho)	CPE_{dl} N	W_D Y_0 (S . $\sqrt{s}$) (mMho)
The unmodified material electrode	941	18.2	0.326	0.908	0.217
CNT/graphene electrode	468	0.788	4.35	0.821	1.80

Table S3 Comparison of analytical recovery studies using our model with a printed bandage-based biosensing array incorporating the pH-correction system and traditional calibration methods using modeled wound fluids at various pH levels.

**Our proposed model**				**Traditional calibration method**			
**Sample**	**Concentration (μM)**	**Detected (μM)**	**%Recovery ± SD (n=3)**	**Sample**	**Concentration (μM)**	**Detected (μM)**	**%Recovery ± SD (n=3)**
pH 6.5	5	5.28	105.16±0.01	pH 6.5	5	5.35	106.91±0.03
	10	10.08	101.53±0.04		10	10.37	107.49±0.03
	20	20.10	101.00±0.06		20	22.31	123.14±0.03
pH 7.0	5	4.88	97.58±0.05	pH 7.0	5	4.98	99.62±0.01
	10	9.82	96.35±0.01		10	9.72	94.43±0.02
	20	19.35	93.55±0.01		20	20.08	100.79±0.02
pH 8.5	5	5.03	100.57±0.06	pH 8.5	5	3.88	77.60±0.01
	10	9.96	99.24±0.04		10	6.73	34.67±0.06
	20	20.68	106.75±0.12		20	14.00	40.00±0.09

Supporting Information Note 1: The peak current value

The active surface areas of the electrodes were determined by analyzing the relationship between the peak current (I_p) and the square root of the scan rate ($v^{1/2}$) using a known concentration of the $K_3[Fe(CN)_6]$ redox probe. This estimation relies on the Randles–Sevcik equation:

$$I_p = 2.69 \times 10^5 n^{3/2} A D_0 C_0 v^{1/2} \qquad \text{(Supporting Equation S1)}$$

Here, I_p represents the peak current (A), n indicates the number of electrons transferred (set to 1 for $K_3[Fe(CN)_6]$), A is the surface area of the electrode (cm²), D_0 is the diffusion coefficient ($D_0 = 7.6\times10^{-6}$ cm² s⁻¹ for $K_3[Fe(CN)_6]$), C_0 is the concentration of the electroactive species (mol cm⁻³), and v is the scan rate (V s⁻¹) [19].

Supporting Information Note 2: The areal capacitance calculations

$$C_{areal} = \left(\frac{1}{2\times(V_2-V_1)\times v}\int_{V_1}^{V_2}|I(V)|dV\right)\frac{1}{A} = \frac{C}{A} \qquad \text{(Supporting Equation S2)}$$

To calculate the specific capacitance (F cm⁻²) from cyclic voltammetry data, where I (V) represent the current at each voltage (A), V_2 and V_1 denote the upper and lower limits of the chosen voltage range (V), v stands for the scan rate (V s⁻¹), and A represents the geometric surface area of the electrode (cm²). Then, the resulting specific capacitance is divided by the area of the working electrode (0.1 cm⁻²) [20].

Supporting Information 3: The surface concentration of the electroactive pyocyanin species

The surface concentration of the electroactive pyocyanin species, denoted as Γ, can be estimated using the following relationship:

$$I_p = n^2 F^2 \frac{A\Gamma v}{4RT} \qquad \text{(Supporting Equation S3)}$$

Here, n represents the number of electrons involved in the oxidation (n = 2), A denotes the geometric surface area of the electrode (0.1 cm²), Γ (in mol cm⁻²) signifies the surface coverage, v denotes the scan rate, F is Faraday's constant (96,485 C mol⁻¹), R is the gas constant (8.314 J K⁻¹ mol⁻¹), and T is the temperature (298.15 K). The surface concentration coverage of pyocyanin can be estimated by utilizing the slope of the anodic peak current versus scan rate [21, 22].

References

1. O. Simoska, J. Duay, K. J. Stevenson. Electrochemical detection of multianalyte biomarkers in wound healing efficacy. ACS Sens. **5**, 3547-3557 (2020). https://doi.org/10.1021/acssensors.0c01697

2. S. Sriwiriyajan, T. Ninpesh, Y. Sukpondma, T. Nasomyon, P. Graidist. Cytotoxicity screening of plants of genus piper in breast cancer cell lines. Tropical Journal of Pharmaceutical Research. **13**, 921 (2014). https://doi.org/10.4314/tjpr.v13i6.14

3. A. Cernat, A. Canciu, M. Tertis, F. Graur, C. Cristea. Synergic action of thermosensitive hydrogel and au/ag nanoalloy for sensitive and selective detection of pyocyanin. Analytical and Bioanalytical Chemistry. **411**, 3829-3838 (2019). https://doi.org/10.1007/s00216-019-01857-4

4. D. Sharp, P. Gladstone, R. B. Smith, S. Forsythe, J. Davis. Approaching intelligent infection diagnostics: Carbon fibre sensor for electrochemical pyocyanin detection. Bioelectrochemistry. **77**, 114-119 (2010). https://doi.org/10.1016/j.bioelechem.2009.07.008

5. F. A. a. Alatraktchi, J. S. Noori, G. P. Tanev, J. Mortensen, M. Dimaki, H. K. Johansen, J. Madsen, S. Molin, W. E. Svendsen. Paper-based sensors for rapid detection of virulence factor produced by pseudomonas aeruginosa. PLOS ONE. **13**, e0194157 (2018). https://doi.org/10.1371/journal.pone.0194157

6. H. Manisha, J. Sonia, S. Shashikiran, S. Yuvarajan, P. D. Rekha, K. Sudhakara Prasad. Computer numerical control-printed paper electrodes for electrochemical detection of pseudomonas aeruginosa virulence factor pyocyanin. Electrochemistry Communications. **137**, 107259 (2022). https://doi.org/10.1016/j.elecom.2022.107259

7. P. Thirabowonkitphithan, P. Phuengmaung, A. Leelahavanichkul, W. Laiwattanapaisal. Mwcnts/pva hydrogel-modified electrochemical sensors for ex vivo and in vivo detection of pyocyanin biomarker for pseudomonas aeruginosa wound infection. ACS Applied Electronic Materials. **5**, 821-831 (2023). https://doi.org/10.1021/acsaelm.2c01396

8. R. Burkitt, D. Sharp. Submicromolar quantification of pyocyanin in complex biological fluids using pad-printed carbon electrodes. Electrochemistry Communications. **78**, 43-46 (2017). https://doi.org/10.1016/j.elecom.2017.03.021

9. F. A. Alatraktchi, S. B. Andersen, H. K. Johansen, S. Molin, W. E. Svendsen. Fast selective detection of pyocyanin using cyclic voltammetry. Sensors (Basel). **16**, (2016). https://doi.org/10.3390/s16030408

10. J. I. A. Rashid, V. Kannan, M. H. Ahmad, A. A. Mon, S. Taufik, A. Miskon, K. K. Ong, N. A. Yusof. An electrochemical sensor based on gold nanoparticles-functionalized reduced graphene oxide screen printed electrode for the detection of pyocyanin biomarker in pseudomonas aeruginosa infection. Materials Science and Engineering: C. **120**, 111625 (2021). https://doi.org/10.1016/j.msec.2020.111625

11. T. A. Webster, E. D. Goluch. Electrochemical detection of pyocyanin in nanochannels with integrated palladium hydride reference electrodes. Lab on a Chip. **12**, 5195-5201 (2012). https://doi.org/10.1039/C2LC40650K

12. P. Thirabowonkitphithan, S. Hajizadeh, W. Laiwattanapaisal, L. Ye. Detection of pseudomonas aeruginosa infection using a sustainable and selective polydopamine-based molecularly imprinted electrochemical sensor. European Polymer Journal. **209**, 112892 (2024). https://doi.org/10.1016/j.eurpolymj.2024.112892

13. L. Liu, X. Cao, W. Ma, L. Chen, S. Li, B. Hu, Y. Xu. In-situ and continuous monitoring of pyocyanin in the formation process of pseudomonas aeruginosa biofilms by an electrochemical biosensor chip. Sensors and Actuators B: Chemical. **327**, 128945 (2021). https://doi.org/10.1016/j.snb.2020.128945

14. J. Elliott, O. Simoska, S. Karasik, J. B. Shear, K. J. Stevenson. Transparent carbon ultramicroelectrode arrays for the electrochemical detection of a bacterial warfare toxin, pyocyanin. Analytical Chemistry. **89**, 6285-6289 (2017). https://doi.org/10.1021/acs.analchem.7b00876

15. A. A. Elkhawaga, M. M. Khalifa, O. El-Badawy, M. A. Hassan, W. A. El-Said. Rapid and highly sensitive detection of pyocyanin biomarker in different pseudomonas aeruginosa infections using gold nanoparticles modified sensor. PLoS One. **14**, e0216438 (2019). https://doi.org/10.1371/journal.pone.0216438

16. A. Buzid, J. Reen, V. Langsi, E. Ó Muimhneacháin, F. O'Gara, G. McGlacken, J. Luong, J. Glennon. Direct and rapid electrochemical detection of pseudomonas aeruginosa quorum signaling molecules in bacterial cultures and cystic fibrosis sputum samples through cationic surfactant-assisted membrane disruption. **4**, (2017). https://doi.org/10.1002/celc.201600590

17. O. Simoska, M. Sans, M. D. Fitzpatrick, C. M. Crittenden, L. S. Eberlin, J. B. Shear, K. J. Stevenson. Real-time electrochemical detection of pseudomonas aeruginosa phenazine metabolites using transparent carbon ultramicroelectrode arrays. ACS Sensors. **4**, 170-179 (2019). https://doi.org/10.1021/acssensors.8b01152

18. Y. Liu, J. Luo, M. Pan, Q. Xu, Z. Liu, B. Liu, S. Zhou, R. Wu. Electrochemical sensing platform constructed from cobalt(ii) tetra-aminophthalocyanine and mxene (ti3c2tx) composites for the detection of pseudomonas aeruginosa biomarkers-pyocyanin. Applied Surface Science. **622**, 156889 (2023). https://doi.org/10.1016/j.apsusc.2023.156889

19. I. H. Hamzah, A. A. Manaf, O. Sidek. A study on characteristic and reliability of fabricated microfluidic three electrodes sensor based on randle-sevcik equation. 2010 IEEE Asia Pacific Conference on Circuits and Systems. 816-819 (2010)

20. N. Rasitanon, W. Sangsudcha, I. Jeerapan. A diaper-based printed sensing array for noninvasively and speedily detecting morphine and potassium ions. Chemical Engineering Journal. 148898 (2024). https://doi.org/10.1016/j.cej.2024.148898

21. M. Sharp, M. Petersson, K. Edström. Preliminary determinations of electron transfer kinetics involving ferrocene covalently attached to a platinum surface. Journal of Electroanalytical Chemistry and Interfacial Electrochemistry. **95**, 123-130 (1979). https://doi.org/10.1016/S0022-0728(79)80227-2

22. X. Lin, Y. Zhang, W. Chen, P. Wu. Electrocatalytic oxidation and determination of dopamine in the presence of ascorbic acid and uric acid at a poly (p-nitrobenzenazo resorcinol) modified glassy carbon electrode. Sensors and Actuators B: Chemical. **122**, 309-314 (2007). https://doi.org/10.1016/j.snb.2006.06.004
